# Effectiveness of angiotensin converting enzyme inhibitors in preventing pneumonia: A systematic review and meta‐analysis

**DOI:** 10.1002/jgf2.532

**Published:** 2022-03-10

**Authors:** Hideki Tsunoda, Yukiko Okami, Yuki Honda, Akihiro Shiroshita, Yuki Kataoka, Yasushi Tsujimoto, Kazuhiro Matsumura

**Affiliations:** ^1^ 13051 Department of Family Medicine Shiga University of Medical Science Otsu Japan; ^2^ Department of Family Medicine University of Pittsburgh Medical Center Shadyside Pittsburgh PA USA; ^3^ Scientific Research Works Peer Support Group (SRWS‐PSG) Osaka Japan; ^4^ NCD Epidemiology Research Center Shiga University of Medical Science Otsu Japan; ^5^ Department of General Internal Medicine Seirei Hamamatsu General Hospital Hamamatsu Japan; ^6^ Department of pulmonology Kameda Medical Center Kamogawa Japan; ^7^ Department of Internal Medicine Kyoto Min‐Iren Asukai Hospital Sakyo‐ku Japan; ^8^ Section of Clinical Epidemiology, Department of Community Medicine Kyoto University Graduate School of Medicine Sakyo‐ku Japan; ^9^ Department of Healthcare Epidemiology Kyoto University Graduate School of Medicine/ School of Public Health Sakyo‐ku Japan; ^10^ Department of Nephrology and Dialysis Kyoritsu Hospital Kawanishi Japan

**Keywords:** angiotensin‐converting enzyme inhibitors, mortality, pneumonia, risk

## Abstract

We performed a systematic review and meta‐analysis to re‐evaluate the effectiveness of angiotensin‐converting enzyme inhibitors (ACE‐I) in the reduction of pneumonia risk. We searched relevant publications in five databases. All studies included patients older than 18 years, who had used ACE‐I as an intervention, and had assessed pneumonia. Seven RCTs (*n* = 8704) and 38 observational studies (*n* = 1,705,030) were included. The overall risk of bias was high. ACE‐I‐treated patients were associated with a slightly lower risk of pneumonia, both from pooled estimates of RCTs [pooled odds ratio (OR), 0.75; 95% confidence interval (CI), 0.62–0.90; low certainty of evidence] and observational studies (pooled OR, 0.85; 95% CI, 0.78–0.92; very low certainty of evidence). Considering the small effect size of ACE‐I in preventing pneumonia and the low quality of the evidence, routine use of ACE‐I for pneumonia prevention is not recommended.

## Graphical abstract

In this study, we performed a systematic review and meta‐analysis of the updated literature to re‐assess whether angiotensin converting enzyme inhibitors (ACE‐I) reduced the risk of pneumonia in adults older than 18 years. We found that ACE‐I were associated with a slightly lower risk of pneumonia, however, considering the small effect size of ACE‐I in preventing pneumonia observed in this review, their routine use for pneumonia prevention may only be recommended in specific populations, such as older adults.

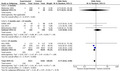

## 1 BACKGROUND

Healthcare providers frequently encounter pneumonia in both outpatient and inpatient settings. Although it is a commonly occurring disease, pneumonia may become life‐threatening, especially for older adults.^1^, ^2^ Lower respiratory tract infections caused approximately 2.4 million deaths worldwide in 2016, and 45% of these deaths were in adults older than 70 years.^3^ In the United States, approximately 44,000 patients died because of pneumonia in 2019,^4^ and pneumonia/influenza was the ninth leading cause of death.^5^ In the United States, pneumonia is responsible for approximately 0.4% of outpatient and emergency room visits annually,^6^ and more than 1.5 million adults are estimated to be admitted to the hospital on account of pneumonia every year.^7^

Given the significant societal effect of this common disease, its prevention is also important. Smoking cessation, influenza vaccination, and pneumococcal vaccination effectively prevent pneumonia.^8^, ^9^, ^10^ Oral care, including brushing and treating poorly maintained teeth, is also believed to be effective because a small amount of aspiration can occur even in a healthy individual; aspiration is considered an important mechanism in the occurrence of pneumonia.^11^

Angiotensin converting enzyme inhibitors (ACE‐I), widely used in hypertension, are believed to protect against pneumonia.^12^ The promotion of cough reflex because of increased substance P and bradykinin is a major adverse effect of ACE‐I therapy and could also be one of the possible mechanisms through which the drug reduces pneumonia risk.^13^, ^14^, ^15^ This preventive effect seems to be pronounced in specific populations, such as patients with a history of stroke in whom cough reflex is expected to be suppressed.^16^, ^17^ A previous systematic review published in 2012 reported that treatment with ACE‐I led to a 34% reduction in pneumonia when compared to control treatment.^12^ However, several new studies have contrasting results.^18^, ^19^, ^20^ Additionally, the articles included in the previous review are now outdated. Therefore, we suspected that ACE‐I might not be as effective in preventing pneumonia as they were thought to be; therefore, we aimed to update the evidence on their effectiveness in preventing pneumonia.

## 2 METHODS

This systematic review was conducted according to the Cochrane Handbook^21^ and recommendations of the Preferred Reporting Items for Systematic Reviews and Meta‐Analyses (PRISMA) main statement^22^ (see the PRISMA checklist in Table S1).

### 2.1 Protocol and registration

The protocol is accessible on the UMIN‐CTR Clinical Trial website (registration number UMIN000039424).

### 2.2 Eligibility criteria

Studies were included regardless of language if they: (1) were randomized controlled trials (RCTs), quasi‐RCTs, and observational studies; (2) used ACE‐I as an intervention for adult patients; and (3) evaluated the risk of pneumonia as an outcome. We included any outcome related to incidence of pneumonia, such as hospitalization because of pneumonia. We excluded studies in which the outcome apparently differed from that of bacterial or aspiration pneumonia, such as radiation pneumonitis. We also excluded studies that addressed the association between ACE‐I use and the prognosis of pneumonia. Regarding observational studies, we included exploratory studies that had ACE‐I as the main exposure or one of the main treatments but excluded the studies that had ACE‐I only as a covariate because collecting the necessary information is difficult in such cases.

We set the incidence of pneumonia, mortality, and withdrawal because of adverse effects (WDAEs) as the primary outcomes, and swallowing function as the secondary outcome. We included these outcomes regardless of the intervention duration and reporting time. We defined the diagnosis of pneumonia as the presence of new pneumonic changes in the chest x‐ray and clinical signs of pneumonia, namely cough, fever, sputum production, and pleuritic chest pain. We also accepted any definition the original authors set. We defined WDAEs as dropout because of any symptoms but did not include withdrawal of consent. We included the outcome of swallowing function if it was assessed using the Royal Brisbane Hospital Outcome Measure for Swallowing (RBHOMS) score,^23^ the dysphagia severity rating scale, functional oral intake scale, and dysphagia outcome and severity scale or when determined using water swallowing tests.

### 2.3 Information sources and search

We searched for studies in the following databases: the Cochrane Central Register of Controlled Trials (CENTRAL), MEDLINE via Ovid, and EMBASE. To identify ongoing and unpublished studies, we explored the World Health Organization International Clinical Trials Platform Search Portal (ICTRP) and ClinicalTrials.gov. We also searched reference lists of the studies included and clinical practice guidelines.^24^, ^25^ A detailed listing of the search terms used in this review are given in Table S2. Briefly, search terms were created by combining the terms “ACE‐Inhibitor” and “pneumonia.” All searches were performed on different days in January and February 2021, and the oldest search was conducted on CENTRAL on January 29, 2021.

### 2.4 Study selection

Two of three researchers (H.T., Y.O., and Y. H) independently assessed the inclusion eligibility of the studies retrieved. The initial selection was based on the title and abstract. When the title and abstract provided insufficient information to determine the relevance, a full‐text copy of the article was retrieved and reviewed. For the final selection, a full‐text copy was examined. Disagreements about inclusion were resolved by discussion. We contacted the original investigators if sufficient information on eligibility was not available in the full text. When no consensus could be reached, another set of researchers (Y.T. and Y.K.) made the final decision.

### 2.5 Data extraction

Data were extracted using a standard data entry form by two of the three aforementioned researchers. We extracted basic characteristics (such as number and characteristics of participants) and information about each outcome. For dichotomous outcomes (pneumonia, mortality, and WDAE), we extracted the number of participants and events in each group and, where possible, the adjusted odds ratio (OR). For swallowing function, we extracted the number of participants, mean, and standard deviation in each group. We also extracted specific information, such as the percentage of history of stroke among participants or ethnicity, for subgroup and sensitivity analyses. Regarding observational studies, we extracted confounders pre‐specified by us. In terms of the outcome of incidence of pneumonia in cohort studies, we calculated the OR using a simple count if the study's observational period was short, even if we were not sure whether the outcome was counted repeatedly. Any disagreement was resolved by discussion and, if needed, a final decision was made by the third and fourth researchers (Y.T. and Y.K.).

### 2.6 Risk of Bias in individual studies

Two of the three researchers carried out risk‐of‐bias (ROB) assessments using the Cochrane Collaboration risk‐of‐bias tool v.2.0 that has the following five domains: randomization, deviation from intervention, missing data, measurement of outcome, and selective reporting.^26^ Each domain and overall were rated “high risk,” “some concerns,” and “low risk.” For observational studies, we used the Newcastle–Ottawa Scale (NOS) with domains for selection, comparability, and exposure/outcome.^27^ Regarding NOS, we assigned (i) age; (ii) diabetes mellitus (DM), chronic kidney disease (CKD), and congestive heart failure (CHF) statuses; and (iii) history of pneumonia as the most important (primary) confounders and (i) sex; (ii) presence of chronic obstructive pulmonary disease (COPD) or asthma; (iii) history of stroke; (iv) ongoing medication (antacid, oral corticosteroid, and other immunosuppressants); (v) smoking status; and (vi) immunization status (against influenza and *Streptococcus pneumonia*) as secondary confounders. We added one point to the score of comparability of NOS if all primary confounders were adjusted for. An additional point was added if all the secondary confounders were also adjusted for. If the Charlson comorbidity index score was used, we considered age, CHF, CKD, DM, history of stroke, COPD, and asthma to be adjusted. We judged that the history of pneumonia was adjusted if the study looked back over the past year.

### 2.7 Synthesis of results

Data were synthesized using the random effects model. We evaluated heterogeneity using I^2^ and tau^2^ statistics and the Cochrane chi^2^ test (*Q*‐test). We performed meta‐analyses separately for randomized trials and observational studies. For dichotomous outcomes, we pooled the OR and its 95% confidence intervals (CIs) as effect size. For integration, we used the Mantel–Haenszel method for RCTs and the generic inverse variance method for observational studies. Regarding observational studies, we used adjusted ORs, if available; if not, we used the crude OR. If only the hazard ratio (HR) was available, we did not use the data in the meta‐analysis but summarized it qualitatively. For continuous outcomes, we pooled the mean difference (MD) and its 95% CIs using the inverse variance method.

### 2.8 Rating the certainty of evidence

The strength of the overall evidence was assessed with the Grading of Recommendations, Assessment, Development and Evaluations (GRADE) tool into “High,” “Moderate,” “Low,” and “Very Low” certainty groups. Evidence from randomized trials started from “High” quality and can be downgraded for risk of bias (as described previously), inconsistency (statistical or theoretical heterogeneity between studies), indirectness (deviation from the population or intervention of review), and other considerations, including publication bias. Evidence from nonrandomized studies started from “Low” quality and can be downgraded as described but can also be upgraded if a large effect size or evidence of dose sensitivity was found or if all plausible remaining confounding would decrease the effect size.

### 2.9 Additional analyses

We performed pre‐specified analyses for incidence of pneumonia in the following subgroups: (i) Study follow‐up: short (<3 months), intermediate (3–24 months), or long term (≥24 months); (ii) race (Asian or not); (iii) older patients (i.e., whether studies included a minority [<20%] of patients aged <65 years); (iv) history of stroke; and (v) presence of neurodegenerative diseases.

We also performed sensitivity analyses for pneumonia incidence by: (i) exclusion of studies using imputed statistics; (ii) exclusion of studies using a diagnostic criteria of pneumonia other than the presence of new pneumonic changes in the chest X‐ray and clinical signs of pneumonia such as cough, fever, sputum production, and pleuritic chest pain; (iii) exclusion of studies which examined for pneumonia only in inpatient or outpatient settings; and (iv) exclusion of studies using the effect estimates from univariate analyses instead of those from multivariable analyses (only for observational studies).

We used the Review Manager version 5.4^28^ and GRADE Pro software^29^ for all analyses.

### 2.10 Difference in the protocol and review

As for a subgroup analysis of Asian patients, we included those studies that used data from Asian countries or that identified non‐Asians as a subgroup in multi‐nation cohorts. We also added sensitivity analysis after the exclusion of studies that examined for pneumonia only in inpatient or outpatient settings to confirm that the effectiveness of ACE‐I in preventing pneumonia does not change in response to the severity of the pneumonia. We could not perform WDAE analysis on the observational studies, and we refrained from completing swallowing function evaluations in both the RCTs and observational studies because of the limited data available. We also could not perform subgroup analyses for patients with a history of stroke or neurodegenerative disease in the RCTs. In addition, we could not complete a sensitivity analysis of the studies that examined pneumonia in both inpatient and outpatient settings because of an insufficient number of eligible studies in the RCTs.

## 3 RESULTS

### 3.1 Study selection

A total of 1962 records were identified from the databases. After adding additional records from citation search and review of bibliographies of relevant guidelines, we identified 98 records. After reviewing the full texts, 46 records describing 45 studies were included; of which seven were randomized trials (*n* = 8704), and 38 were observational studies (*n* = 1,705,030) (Figure S1). Detailed citations for the RCTs and observational studies can be found in Table S3, and the information of the excluded studies can be found in Table S4. One of the observational studies used five different data sources, and we dealt with this study as five different studies.^30^

### 3.2 Study characteristics

Regarding RCTs, the range of sample size varied from 93 to 6105 participants, and the mean follow‐up length was from approximately 2 months to 3.9 years. Only two of seven studies investigated the risk of pneumonia as the primary outcome for patients who were at high risk of aspiration, such as patients with a history of stroke or those who were tube‐fed. The other five studies were conducted to prove the effectiveness of ACE‐I in certain populations, such as patients with chronic heart failure or CKD, and reported pneumonia as an adverse event. One study used angiotensin II receptor blockers (ARBs) as the control, and the others used placebos.

Regarding observational studies, 22 were cohort and 16 were case–control studies, and the sample size ranged from 73 to 1,339,169. Among the 22 cohort studies, 12 were published after 2014, and most were retrospective, using big data such as claim data or regional population databases. Contrastingly, the remaining 10 studies published before 2014 were mainly prospective and used the local data of certain facilities. In the cohort studies, 13 included patients treated with hypertensive medications other than ACE‐I as control, whereas others included nonrecipients of ACE‐I as controls. Eleven studies reported the risk of pneumonia as adjusted HRs and the other studies reported adjusted or crude ORs. Additional details of the 45 included studies, including characteristics of the study participants, are shown in Tables S5–S7.

### 3.3 Risk of bias within studies

Regarding RCTs, ROB was described for the seven randomized trials (Tables S8–S11). For the risk of pneumonia, we judged only one of the seven studies as having low and the rest (*n* = 6) as having high overall ROB. The main reason for the high overall ROB was bias in the measurement of the outcome; five of the seven randomized studies reported the outcome of pneumonia as an adverse effect, and the definition of pneumonia was unclear. Regarding observational studies, the NOS score (Tables S12–S14) of most studies (except three) were ≤5 points for pneumonia risk. The quality, based on overall ROB depending on the total NOS score, of most of the observational studies were judged as poor mainly because of insufficient adjustment of the confounding factors.^31^ For mortality, four of seven randomized studies had low ROB and the rest had some concerns or high ROB. In cohort studies, the quality was good only in one study and poor in the other three. For the risk of WDAE and swallowing function, all studies had high ROB.

### 3.4 Results of individual studies and synthesis of results

The summary of findings can be found in Tables 1 and 2.

**TABLE 1:** Summary of findings: A comparison between angiotensin converting enzyme inhibitor treatment and control treatment (randomized control trials)

Patient or population: Adults, Setting: Anywhere, Intervention: ACE‐I, Comparison: Control treatment					
Outcomes	Anticipated absolute effects^a^ (95% CI)		Relative effect (95% CI)	No. of participants (studies)	Certainty of the evidence (GRADE)
	Risk with placebo	Risk with ACE‐I			
Risk of pneumonia	69 per 1,000	52 per 1000 (44–62)	OR 0.75 (0.62–0.90)	8704 (7 RCTs)	⨁⨁◯◯ Low^a^
Mortality	165 per 1,000	165 per 1000 (117–226)	OR 1.00 (0.67–1.48)	8704 (7 RCTs)	⨁⨁◯◯ Low^a^
Withdrawal because of adverse effects	20 per 1,000	49 per 1000 (27–88)	OR 2.51 (1.35–4.68)	6601 (6 studies)	⨁⨁◯◯ Low^a^
Swallowing function	The mean swallowing function measured by The Royal Brisbane Hospital Outcome Measure for Swallowing score was 4.2	MD 0.7 higher (−0.16 lower to 1.56 higher)	–	48 (1 RCT)	⨁◯◯◯ Very low^a^

Abbreviations: ACE‐I, angiotensin converting enzyme inhibitor; CI, Confidence interval; GRADE, Grading of Recommendations, Assessment, Development and Evaluations; MD, mean difference; OR, Odds ratio; RCT, randomized control trial.

**GRADE Working Group grades of evidence**

High certainty: We are very confident that the true effect lies close to that of the estimate of the effect.

Moderate certainty: We are moderately confident in the effect estimate: the true effect is likely to be close to the estimate of the effect, but there is a possibility that it is substantially different.

Low certainty: Our confidence in the effect estimate is limited: the true effect may be substantially different from the estimate of the effect.

Very low certainty: We have very little confidence in the effect estimate: the true effect is likely to be substantially different from the estimate of effect.

**Explanation**

a. Downgraded because of risk of bias. (GRADE of the outcome of Swallowing function was downgraded by second degree because of risk of bias).

b. Downgraded because of publication bias.

c. Downgraded because of heterogeneity in the result.

d. Downgraded because of imprecision (Optimal information size criteria was not met).

The risk in the intervention group (and its 95% confidence interval) is based on the assumed risk in the comparison group and the relative effect of the intervention (and its 95% CI).

**TABLE 2:** Summary of findings: A comparison between angiotensin converting enzyme inhibitor treatment and control treatment (cohort and case–control studies)

Patient or population: Adults, Setting: Anywhere, Intervention: ACE‐I, Comparison: Control treatment					
Outcomes	Anticipated absolute effects^c^ (95% CI)		Relative effect (95% CI)	No. of participants (studies)	Certainty of the evidence (GRADE)
	Risk with placebo	Risk with ACE‐I			
Risk of pneumonia	115 per 1,000^d^	99 per 1,000 (92 to 107)^d^	OR 0.85 (0.78 to 0.92)	60,832 cases/272,584 controls, 1,178,746 exposed /192,868 unexposed (34 observational studies)^e^	⨁◯◯◯ Very low^[Link]^, ^[Link]^
Mortality	113 per 1,000	177 per 1,000 (168 to 184)	OR 1.43 (0.97 to 2.09)	1,170,449 exposed/183,144 unexposed (4 observational studies)	⨁◯◯◯ Very low^b^
Withdrawal because of adverse effects	Not reported	Not reported	Not reported	Not reported	Not reported
Swallowing function	Not reported	Not reported	Not reported	Not reported	Not reported

Abbreviations: ACE‐I, angiotensin converting enzyme inhibitor; CI, Confidence interval; GRADE, Grading of Recommendations, Assessment, Development and Evaluations; OR, Odds ratio.

**GRADE Working Group grades of evidence**

High certainty: We are very confident that the true effect lies close to that of the estimate of the effect.

Moderate certainty: We are moderately confident in the effect estimate: The true effect is likely to be close to the estimate of the effect, but there is a possibility that it is substantially different.

Low certainty: Our confidence in the effect estimate is limited: The true effect may be substantially different from the estimate of the effect.

Very low certainty: We have very little confidence in the effect estimate: The true effect is likely to be substantially different from the estimate of effect.

**Explanation**

Downgraded because of heterogeneity in the result.

Downgraded because of low NOS score.

The risk in the intervention group (and its 95% CI) is based on the assumed risk in the comparison group and the relative effect of the intervention (and its 95% CI).

We included only cohort studies and excluded the following studies because the risk is not available: Bang 2015, Ishifuji 2017.

We excluded the following study because the number of participants is not available: Bang 2015.

### 3.5 Risk of pneumonia

**FIGURE 1:**
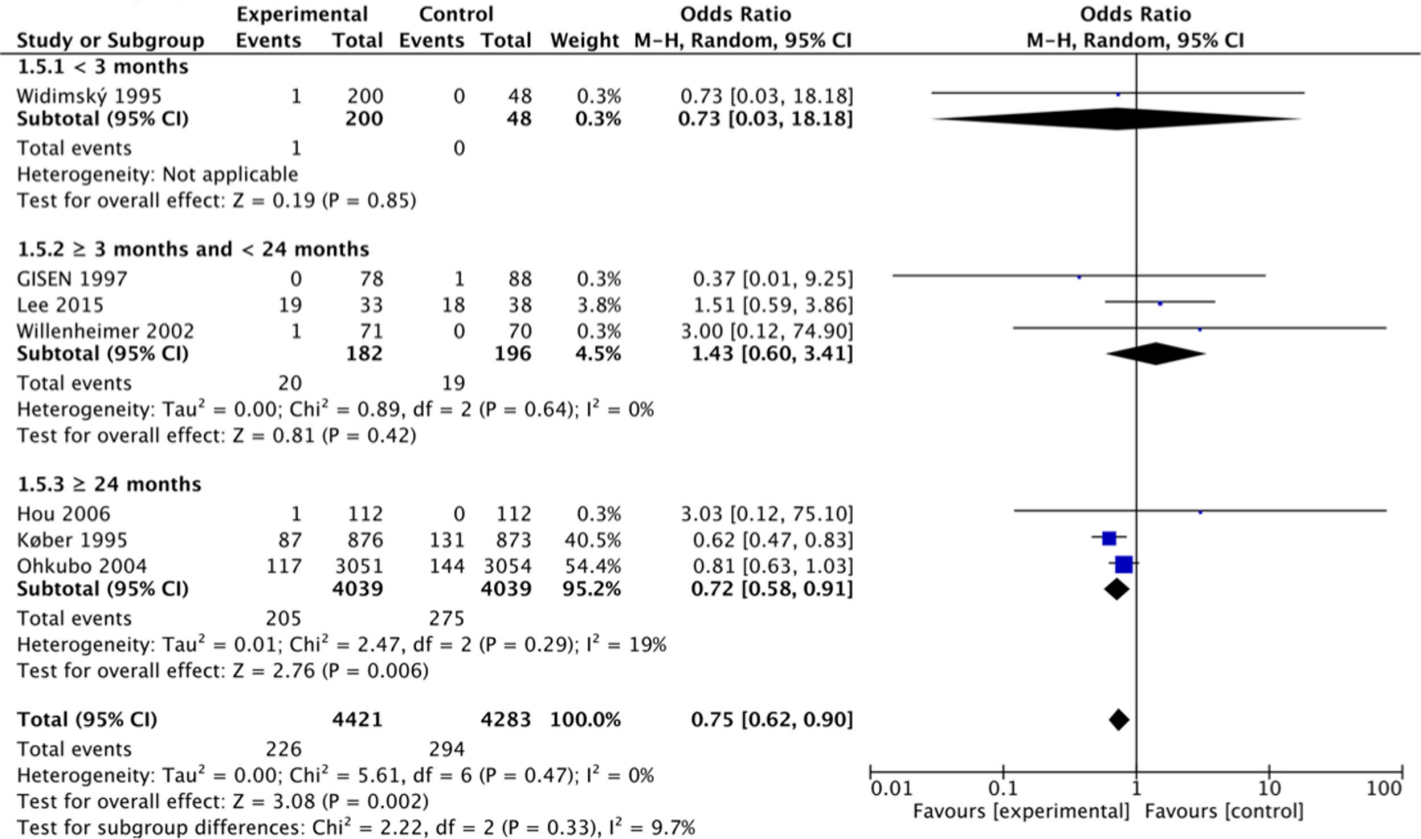
Risk of pneumonia with use of angiotensin converting enzyme inhibitors (ACE‐I) compared with control treatment among randomized controlled trials. CI, confidence interval

**FIGURE 2:**
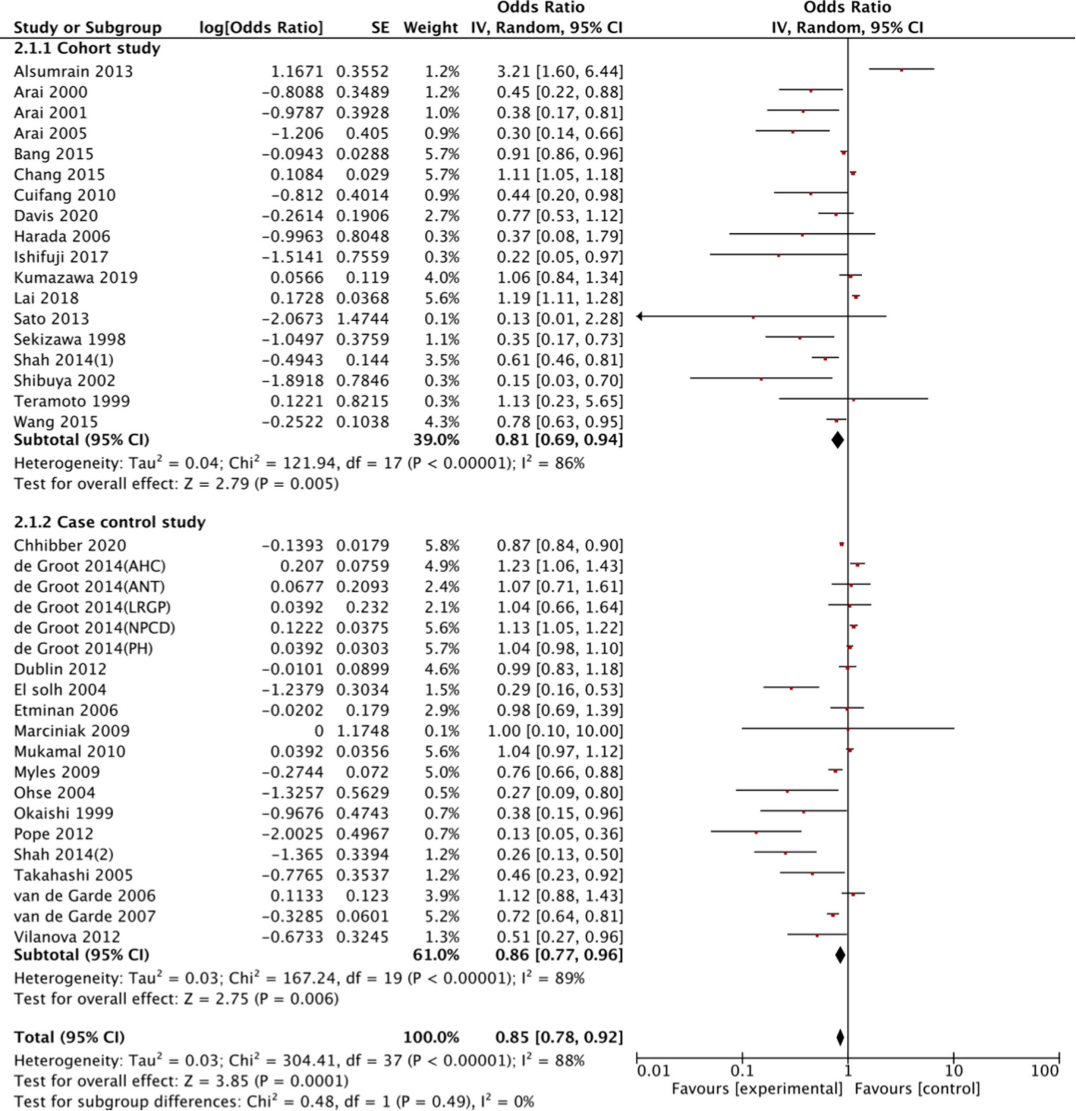
Risk of pneumonia with use of angiotensin converting enzyme inhibitors (ACE‐I) compared with control treatment among cohort and case–control studies; CI, confidence interval; SE, standard error

In total, seven RCTs and 38 observational studies evaluated the effect of ACE‐I on the risk of pneumonia. Regarding RCTs, the range of ORs varied from 0.37 to 3.03 (Figure 1). We synthesized the results of the seven studies using the OR. ACE‐I may reduce the risk of pneumonia slightly (seven studies, 8704 participants: OR, 0.75; 95% CI: 0.62–0.90; *I* ^2^ = 0%; low certainty evidence) (Figure 1 and Table 1). Regarding observational studies, the ORs ranged from 0.13 to 3.21 (Figure 2). The results obtained were not consistent. We synthesized 34 studies in which the OR was available. The meta‐analysis from the observational studies showed a similar result, but the evidence is uncertain regarding the effect of ACE‐I on pneumonia risk (34 studies, 1,705,030 participants; OR, 0.85; 95% CI: 0.78–0.92; *I* ^2^ = 88%; low certainty evidence) (Figure 2 and Table 2). The range of adjusted HRs in the four observational studies that only reported HRs was 0.83–0.89.^32^, ^33^, ^34^, ^35^

### 3.6 Mortality

Seven randomized trials and four observational studies evaluated the effect of ACE‐I on mortality. Regarding RCTs, the range of ORs varied from 0.33–5.33 (Figure S2). The evidence suggests that ACE‐I does not reduce the outcome (seven studies, 8,704 participants; OR, 1.00; 95% CI: 0.67–1.48; *I* ^2^ = 70%; low certainty evidence) (Figure S2 and Table 1). Regarding observational studies, the range of ORs was 0.84–2.28 (Figure S3). The results obtained were discordant, and the evidence is uncertain about the effect of ACE‐I on the outcome (four studies, 1,353,593 participants; OR, 1.43; 95% CI: 0.97–2.09; *I* ^2^ = 96%; very low certainty evidence) (Figure S3 and Table 2).

### 3.7 Withdrawal because of adverse effect

Six randomized studies evaluated the effects of ACE‐I on the risk of WDAE; however, no observational studies related to this outcome were identified. Regarding RCTs, the range of ORs was 0.77–5.35 (Figure S4). ACE‐I may increase the risk of WDAE (six studies, 6,601 participants: OR, 2.51; 95% CI: 1.35–4.68; I^2^=64%; low certainty evidence) (Figure S4 and Table 1).

### 3.8 Swallowing function

Only one randomized study evaluated the effect of ACE‐I on swallowing function with RBHOMS scores. It suggested that ACE‐I may improve the MD of the RBHOMS score a little or cause no change; however, the evidence is very uncertain (one study, 48 participants; MD of the RBHOMS score was 0.7 higher in the intervention group, 95% CI: −0.16–1.56) (Table 1). No observational studies related to this outcome were identified.

#### 3.8.1 Additional analysis

##### Subgroup analyses

There was no heterogeneity in RCTs pertaining to the subgroups of the study period, Asian race, and age (Figure 1 and Table S15). In contrast, regarding observational studies, we found statistical heterogeneity in the subgroups of observational periods, Asian race (as compared with non‐Asian), and older patients (as compared with patients ≤65 years old) (Table S15). There was no significant heterogeneity regarding history of stroke and neurodegenerative disease in the observational studies (Table S15).

##### Sensitivity analysis

Regarding RCTs, after exclusion of the studies using imputation, the significance of the OR disappeared (OR: 0.82; 95% CI: 0.47–1.44), and sensitivity analysis after exclusion of the studies using inappropriate definition of pneumonia showed a contrasting result (Table S15). Regarding observational studies, after exclusion of univariate studies, the significance of the OR disappeared (OR: 0.92; 95% CI: 0.84–1.01). However, other analyses did not show a change in the result (Table S15).

## 4 DISCUSSION

### 4.1 Summary of evidence

We found 45 studies that investigated the efficacy and effectiveness of ACE‐I in preventing pneumonia. For RCTs, ACE‐I may reduce the risk of pneumonia slightly. However, for observational studies, the evidence was very uncertain. ACE‐I therapy may result in an increase in WDAE compared with the control treatment.

Considering the small effect size of ACE‐I in preventing pneumonia, their routine use for this purpose is not recommended. In the previous systematic review, the ORs of ACE‐I for preventing pneumonia were estimated as 0.69 in RCTs, 0.58 in cohort studies, and 0.66 in case–control studies.^12^ However, our review revealed that ACE‐I were less efficacious in preventing pneumonia. A possible explanation for this result is that our study's proportion of elderly or Asian patients was smaller. Our study showed that ACE‐I was more effective in preventing pneumonia in those populations. Hence, the smaller proportion might lower the total odds of pneumonia occurring in the ACE‐I group compared to the non‐ACE‐I group. However, because of the low to the low certainty of the evidence, we could not clearly explain this. Besides, this study did not show significance in mortality. A feasible explanation for this result is that our study focused on studies that investigated mortality in at‐risk populations while the previous review included studies that investigated prognosis in pneumonia patients. We believe that focusing on more at‐risk populations is valuable from a disease prevention perspective. Considering these results and the fact that renin‐angiotensin system (RAS) inhibitors are known to have some serious adverse effects, such as hyperkalemia and acute kidney injury,^36^ our results do not seem to warrant the routine use of ACE‐I exclusively for preventing pneumonia.

Contrastingly, if patients are already on other antihypertensives or have been prescribed antihypertensive medication for the first time, switching to ACE‐I or starting treatment with ACE‐I while expecting the small pneumonia preventive effect adjunctively may be feasible. In standard clinical guidelines for the management of arterial hypertension,^24^, ^25^ ACE‐I are widely used as the first line agents for patients with hypertension, except for those who are pregnant, have renal failure because of bilateral renal artery stenosis, or have a history of angioedema with ACE‐I use.^24^ For elderly or Asian patients requiring antihypertensives, choosing ACE‐I over other hypertensives may be reasonable, barring any contraindications.

It may also be acceptable to prescribe ACE‐I rather than ARBs for patients for whom RAS inhibitors are indicated while expecting a small pneumonia preventive effect. RAS inhibitors are also recommended for patients with heart failure, coronary artery disease, and CKD, especially because of diabetic nephropathy.^37^, ^38^, ^39^ Compared with ARBs, ACE‐I have more solid evidence of efficacy but are more associated with cough and angioedema, resulting in an increase in WDAEs,^36^ which is consistent with the result of this review. Evaluation of the superiority between ACE‐I and ARB is not conclusive for these reasons; the use of ACE‐I rather than ARB might be beneficial, especially for patients who are Asian or elderly.

Finally, it should be noted that the certainty of evidence in our study was low to very low. In RCTs, GRADE was downgraded because of the high ROB and imprecision. In most RCTs, bias in measurement of the outcome was high because the definition and diagnosis of pneumonia were unclear. Since we narrowed down our search by adding the medical subject heading (MeSH) term “pneumonia,” the word relevant to the outcome, it may have resulted in a publication bias. Hence, we downgraded the GRADE score which may have led to lower comprehensiveness. In observational studies, GRADE was downgraded because of low NOS scores and results heterogeneity. Regarding the NOS score, adjustment of confounders was not sufficient, and assessment of dropout of the cohort was rarely described. Moreover, the direction of outcome was not consistent between studies. Further large, well‐designed RCTs will improve the evidence, especially if diagnostic criteria for the definition of pneumonia can help us understand the effect of ACE‐I in preventing pneumonia.

### 4.2 Strengths and Limitations

#### 4.2.1 Strengths

We collected articles exhaustively on the effectiveness of ACE‐I in preventing pneumonia according to the manual of Cochrane Handbook and GRADE system. We added the data of one RCT and 22 observational studies to the previous systematic review^12^ and updated the information. Among the 22 cohort studies, 12 were studies using big data, while the previous review mainly included prospective studies that used the local data of certain facilities. Interestingly, this study revealed a lower effectiveness of ACE‐I in preventing pneumonia compared to the previous study. We did not find a significant difference in subgroup analysis for patients with and without stroke, whereas the previous study did show a significant difference. Moreover, ACE‐I also did not reduce the mortality in the ACE‐I group in observational studies. We consider this result to be meaningful as it suggests that the routine use of ACE‐I to prevent pneumonia should not be recommended, given the limited supporting evidence and minimal effect, which the previous study did not mention.

#### 4.2.2 Limitations

There are several limitations to this study. First, as aforementioned, we narrowed our search strategy by adding the MeSH term “pneumonia,” which may have led to publication bias. We may have overestimated the effectiveness of ACE‐I in preventing pneumonia by selecting articles with better outcomes. However, that does not affect the results of this review. Second, the method of outcome measurement was not consistent between studies, especially in cohort studies, and should be considered while analyzing the results of this review. The studies occasionally reported their results with HR only and adjusted OR was not available; if follow‐up was terminated when the event occurred or the follow‐up time was short, we integrated the results using the crude OR. However, this may have skewed the results of each study. We postulated that this increased the ROB and, hence, lowered the results' certainty of evidence. Third, we could find only one study which investigated the association between swallowing function and ACE‐I therapy, and the evidence was very uncertain; further analysis is required in this topic, which is the most plausible mechanism of how ACE‐I reduce pneumonia.

## 5 CONCLUSIONS

Based on the limited evidence and its minimal effect, routine use of ACE‐I to prevent pneumonia may not be recommended. However, for Asian elderly patients who are already on antihypertensives or those with an indication for RAS inhibitors rather than ARBs, clinicians may consider prescribing ACE‐I for the antihypertensive therapy.

## CONFLICT OF INTEREST

The authors have stated explicitly that there are no conflicts of interest in connection with this article.

## PRIOR PRESENTATIONS

None.

## Supporting information

App S1
