# Supplementary material for: Effectiveness of angiotensin converting enzyme inhibitors in preventing pneumonia: A systematic review and meta‐analysis
Source: J Gen Fam Med. 2022 Mar 10;23(4):217–27. doi: 10.1002/jgf2.532 (PMC9249936; doi:10.1002/jgf2.532)

**Supplementary Table S1: PRISMA checklist**

| **Section/topic** | **#** | **Checklist item** | **Reported on page #** |
| --- | --- | --- | --- |
| **TITLE** | | |  |
| Title | 1 | Identify the report as a systematic review, meta-analysis, or both. | Title Page 1 |
| **ABSTRACT** | | |  |
| Structured summary | 2 | Provide a structured summary including, as applicable: background; objectives; data sources; study eligibility criteria, participants, and interventions; study appraisal and synthesis methods; results; limitations; conclusions and implications of key findings; systematic review registration number. | 1 |
| **INTRODUCTION** | | |  |
| Rationale | 3 | Describe the rationale for the review in the context of what is already known. | 2, 3 |
| Objectives | 4 | Provide an explicit statement of questions being addressed with reference to participants, interventions, comparisons, outcomes, and study design (PICOS). | 3 |
| **METHODS** | | |  |
| Protocol and registration | 5 | Indicate if a review protocol exists, if and where it can be accessed (e.g., Web address), and, if available, provide registration information including registration number. | 3 |
| Eligibility criteria | 6 | Specify study characteristics (e.g., PICOS, length of follow-up) and report characteristics (e.g., years considered, language, publication status) used as criteria for eligibility, giving rationale. | 3, 4, 5 |
| Information sources | 7 | Describe all information sources (e.g., databases with dates of coverage, contact with study authors to identify additional studies) in the search and date last searched. | 5, 6 |
| Search | 8 | Present full electronic search strategy for at least one database, including any limits used, such that it could be repeated. | Supplementary Table S2 |
| Study selection | 9 | State the process for selecting studies (i.e., screening, eligibility, included in systematic review, and, if applicable, included in the meta-analysis). | 5, 6 |
| Data collection process | 10 | Describe method of data extraction from reports (e.g., piloted forms, independently, in duplicate) and any processes for obtaining and confirming data from investigators. | 6 |
| Data items | 11 | List and define all variables for which data were sought (e.g., PICOS, funding sources) and any assumptions and simplifications made. | 6, 7 Supplementary Table S5–7 |
| Risk of bias in individual studies | 12 | Describe methods used for assessing risk of bias of individual studies (including specification of whether this was done at the study or outcome level), and how this information is to be used in any data synthesis. | 7 |
| Summary measures | 13 | State the principal summary measures (e.g., risk ratio, difference in means). | 6 |
| Synthesis of results | 14 | Describe the methods of handling data and combining results of studies, if done, including measures of consistency (e.g., I^2^) for each meta-analysis. | 8 |

| **Section/topic** | **#** | **Checklist item** | **Reported on page #** |
| --- | --- | --- | --- |
| Risk of bias across studies | 15 | Specify any assessment of risk of bias that may affect the cumulative evidence (e.g., publication bias, selective reporting within studies). | 8, 9 |
| Additional analyses | 16 | Describe methods of additional analyses (e.g., sensitivity or subgroup analyses, meta-regression), if done, indicating which were pre-specified. | 9 |
| **RESULTS** | | |  |
| Study selection | 17 | Give numbers of studies screened, assessed for eligibility, and included in the review, with reasons for exclusions at each stage, ideally with a flow diagram. | 10, 11  Supplementary Figure S1  Supplementary Table S3 |
| Study characteristics | 18 | For each study, present characteristics for which data were extracted (e.g., study size, PICOS, follow-up period) and provide the citations. | 11, 12  Supplementary Table S5–7 |
| Risk of bias within studies | 19 | Present data on risk of bias of each study and, if available, any outcome level assessment (see item 12). | 12  Supplementary Table S8–14 |
| Results of individual studies | 20 | For all outcomes considered (benefits or harms), present, for each study: (a) simple summary data for each intervention group (b) effect estimates and confidence intervals, ideally with a forest plot. | 12–14  Figure 1,2  Supplementary Figure S2–4 |
| Synthesis of results | 21 | Present results of each meta-analysis done, including confidence intervals and measures of consistency. | 12–14  Figure 1,2  Supplementary Figure S2–4 |
| Risk of bias across studies | 22 | Present results of any assessment of risk of bias across studies (see Item 15). | Table 1,2 |
| Additional analysis | 23 | Give results of additional analyses, if done (e.g., sensitivity or subgroup analyses, meta-regression [see Item 16]). | 15  Supplementary Table S15 |
| **DISCUSSION** | | |  |
| Summary of evidence | 24 | Summarize the main findings including the strength of evidence for each main outcome; consider their relevance to key groups (e.g., healthcare providers, users, and policy makers). | 15–18 |
| Limitations | 25 | Discuss limitations at study and outcome level (e.g., risk of bias), and at review-level (e.g., incomplete retrieval of identified research, reporting bias). | 19, 20 |
| Conclusions | 26 | Provide a general interpretation of the results in the context of other evidence, and implications for future research. | 20 |
| **FUNDING** | | |  |
| Funding | 27 | Describe sources of funding for the systematic review and other support (e.g., supply of data); role of funders for the systematic review. | Title page |

**Supplementary Table S2: Search Strategies**

| **CENTRAL**  **(31 records found on February 18^th^, 2021)** | 1. [mh "Angiotensin-Converting Enzyme Inhibitors"] OR [mh captopril] OR [mh cilazapril] OR [mh enalapril] OR [mh enalaprilat] OR [mh fosinopril] OR [mh lisinopril] OR [mh perindopril] OR [mh quinapril] OR [mh ramipril] OR [mh teprotide] OR alacepril:kw OR aladotril:kw OR ancovenin:kw OR "AVE 7688":kw OR benazepril:kw OR benazeprilat:kw OR ceronapril:kw OR "CGS 13945":kw OR cilazaprilat:kw OR desacetyl-alacepril:kw OR delapril:kw OR foroxymithine:kw OR gemopatrilat:kw OR idrapril:kw OR imidapril:kw OR Indolapril:kw OR libenzapril:kw OR moexipril:kw OR omapatrilat:kw OR perindoprilat:kw OR quinaprilat:kw OR ramiprilat:kw OR rentiapril:kw OR sampatrilat:kw OR S-nitrosocaptopril:kw OR spirapril:kw OR "temocapril hydrochloride":kw OR trandolapril:kw OR utibapril:kw OR zabicipril:kw OR zofenopril:kw 2. "angiotensin converting enzyme inhibitor":ti,ab OR "angiotensin converting enzyme inhibitors":ti,ab OR acei*:ti,ab OR "ace inhibitor*":ti,ab OR alacepril:ti,ab OR aladotril:ti,ab OR altiopril:ti,ab OR ancovenin:ti,ab OR "AVE 7688":ti,ab OR benazepril*:ti,ab OR captopril:ti,ab OR ceranapril:ti,ab OR ceronapril:ti,ab OR "CGS 13945":ti,ab OR cilazapril*:ti,ab OR deacetylalacepril:ti,ab OR desacetyl-alacepril:ti,ab OR delapril:ti,ab OR derapril:ti,ab OR enalapril*:ti,ab OR epicaptopril:ti,ab OR fasidotril*:ti,ab OR fosinopril:ti,ab OR "fosinoprilic acid":ti,ab OR foroxymithine:ti,ab OR gemopatrilat:ti,ab OR idrapril:ti,ab OR ilepatril:ti,ab OR imidapril*:ti,ab OR indolapril:ti,ab OR libenzapril:ti,ab OR lisinopril:ti,ab OR moexipril*:ti,ab OR omapatrilat:ti,ab OR pentopril*:ti,ab OR perindopril*:ti,ab OR pivopril:ti,ab OR quinapril*:ti,ab OR ramipril*:ti,ab OR ramiprilat:ti,ab OR rentiapril:ti,ab OR sampatrilat:ti,ab OR "s nitrosocaptopril":ti,ab OR S-nitrosocaptopril:ti,ab OR spirapril*:ti,ab OR temocapril*:ti,ab OR "temocapril hydrochloride":ti,ab OR teprotide:ti,ab OR trandolapril*:ti,ab OR utibapril*:ti,ab OR zabicipril*:ti,ab OR zofenopril*:ti,ab OR aceon:ti,ab OR accupril:ti,ab OR altace:ti,ab OR capoten:ti,ab OR lotensin:ti,ab OR mavik:ti,ab OR monopril:ti,ab OR prinivil:ti,ab OR univas:ti,ab OR vasotec:ti,ab OR zestril:ti,ab 3. [mh "Pneumonia, Aspiration"] OR pneumon*:ti,ab 4. [mh animals] NOT [mh humans] 5. **1 OR 2** 6. 5 AND 3 7. 6 NOT 4 |
| --- | --- |
| **MEDLINE (via PubMed)**  **( 271 records found on January 27^th^, 2021)** | 1. **"Angiotensin-Converting Enzyme Inhibitors"[mh] OR captopril[mh] OR cilazapril[mh] OR enalapril[mh] OR enalaprilat[mh] OR fosinopril[mh] OR lisinopril[mh] OR perindopril[mh] OR quinapril[mh] OR ramipril[mh] OR teprotide[mh] OR alacepril[nm] OR aladotril[nm] OR ancovenin[nm] OR "AVE 7688"[nm] OR benazepril[nm] OR benazeprilat[nm] OR ceronapril[nm] OR "CGS 13945"[nm] OR cilazaprilat[nm] OR desacetyl-alacepril[nm] OR delapril[nm] OR foroxymithine[nm] OR gemopatrilat[nm] OR idrapril[nm] OR imidapril[nm] OR Indolapril[nm] OR libenzapril[nm] OR moexipril[nm] OR omapatrilat[nm] OR perindoprilat[nm] OR quinaprilat[nm] OR ramiprilat[nm] OR rentiapril[nm] OR sampatrilat[nm] OR S-nitrosocaptopril[nm] OR spirapril[nm] OR temocapril hydrochloride[nm] OR trandolapril[nm] OR utibapril[nm] OR zabicipril[nm] OR zofenopril[nm]** 2. **"angiotensin converting enzyme inhibitor"[tiab] OR "angiotensin converting enzyme inhibitors"[tiab] OR acei*[tiab] OR "ace inhibitor*"[tiab] OR alacepril[tiab] OR aladotril[tiab] OR altiopril[tiab] OR ancovenin[tiab] OR AVE 7688[tiab] OR benazepril*[tiab] OR captopril[tiab] OR ceranapril[tiab] OR ceronapril[tiab] OR CGS 13945[tiab] OR cilazapril*[tiab] OR deacetylalacepril[tiab] OR desacetyl-alacepril[tiab] OR delapril[tiab] OR derapril[tiab] OR enalapril*[tiab] OR epicaptopril[tiab] OR fasidotril*[tiab] OR fosinopril[tiab] OR fosinoprilic acid[tiab] OR foroxymithine[tiab] OR gemopatrilat[tiab] OR idrapril[tiab] OR ilepatril[tiab] OR imidapril*[tiab] OR indolapril[tiab] OR libenzapril[tiab] OR lisinopril[tiab] OR moexipril*[tiab] OR omapatrilat[tiab] OR pentopril*[tiab] OR perindopril*[tiab] OR pivopril[tiab] OR quinapril*[tiab] OR ramipril*[tiab] OR ramiprilat[tiab] OR rentiapril[tiab] OR sampatrilat[tiab] OR "s nitrosocaptopril"[tiab] OR S-nitrosocaptopril[tiab] OR spirapril*[tiab] OR temocapril*[tiab] OR temocapril hydrochloride[tiab] OR teprotide[tiab] OR trandolapril*[tiab] OR utibapril*[tiab] OR zabicipril*[tiab] OR zofenopril*[tiab] OR aceon[tiab] OR accupril[tiab] OR altace[tiab] OR capoten[tiab] OR lotensin[tiab] OR mavik[tiab] OR monopril[tiab] OR prinivil[tiab] OR univas[tiab] OR vasotec[tiab] OR zestril[tiab]** 3. "Pneumonia, Aspiration"[mh] OR pneumon*[tiab] 4. animals[mh] NOT humans[mh] 5. **1 OR 2** 6. 5 AND 3 7. 6 NOT 4 |
| **EMBASE**  **(1150 records found on January 29^th^, 2021)** | 1. 'angiotensin-converting enzyme inhibitors'/exp OR ’captopril'/exp OR cilazapril'/exp OR ‘enalapril'/exp OR ’enalaprilat'/exp OR fosinopril'/exp OR ‘lisinopril'/exp OR ’perindopril'/exp OR ‘quinapril'/exp OR ‘ramipril'/exp OR ‘teprotide'/exp OR ’alacepril':ab,ti OR ‘cilazaprilat':ab,ti OR desacetyl-alacepril':ab,ti OR ‘delapril':ab,ti OR ‘foroxymithine':ab,ti OR ’gemopatrilat':ab,ti OR ‘idrapri':ab,ti OR ’imidapril':ab,ti OR ’indolapril':ab,ti OR ‘libenzapril':ab,ti OR ’moexipril':ab,ti OR ‘omapatrilat':ab,ti OR ‘perindoprilat':ab,ti OR ’quinaprilat':ab,ti OR ‘ramiprilat':ab,ti OR ‘rentiapril':ab,ti OR ’sampatrilat':ab,ti OR s-nitrosocaptopril':ab,ti OR ‘spirapril':ab,ti OR ‘temocapril hydrochloride':ab,ti OR ‘trandolapril':ab,ti OR ’utibapril':ab,ti OR zabicipril':ab,ti OR ’zofenopril':ab,ti 2. ’angiotensin converting enzyme inhibitor':ti,ab OR ’angiotensin converting enzyme inhibitors':ti,ab OR acei*:ti,ab OR ’ace inhibitor*':ti,ab OR alacepril:ti,ab OR aladotril:ti,ab OR altiopril:ti,ab OR ancovenin:ti,ab OR ‘ave 7688':ti,ab OR benazepril*:ti,ab OR captopril:ti,ab OR ceranapril:ti,ab OR ceronapril:ti,ab OR ‘cgs OR 13945':ti,ab OR cilazapril*:ti,ab OR deacetylalacepril:ti,ab OR ’desacetyl alacepril':ti,ab OR delapril:ti,ab OR derapril:ti,ab OR enalapril*:ti,ab OR epicaptopril:ti,ab OR fasidotril*:ti,ab OR fosinopril:ti,ab OR ‘fosinoprilic acid':ti,ab OR foroxymithine:ti,ab OR gemopatrilat:ti,ab OR idrapril:ti,ab OR ilepatril:ti,ab OR imidapril*:ti,ab OR indolapril:ti,ab OR libenzapril:ti,ab OR lisinopril:ti,ab OR moexipril*:ti,ab OR omapatrilat:ti,ab OR pentopril*:ti,ab OR perindopril*:ti,ab OR pivopril:ti,ab OR quinapril*:ti,ab OR ramipril*:ti,ab OR ramiprilat:ti,ab OR rentiapril:ti,ab OR sampatrilat:ti,ab OR ’s nitrosocaptopril':ti,ab OR ’s-nitrosocaptopril':ti,ab OR spirapril*:ti,ab OR temocapril*:ti,ab OR ‘temocapril hydrochloride':ti,ab OR teprotide:ti,ab OR trandolapril*:ti,ab OR utibapril*:ti,ab OR zabicipril*:ti,ab OR zofenopril*:ti,ab OR aceon:ti,ab OR accupril:ti,ab OR altace:ti,ab OR capoten:ti,ab OR lotensin:ti,ab OR mavik:ti,ab OR monopril:ti,ab OR prinivil:ti,ab OR univas:ti,ab OR vasotec:ti,ab OR zestril:ti,ab 3. pneumon*:ti,ab OR 'pneumonia, aspiration'/exp 4. 'animals'/exp NOT 'humans'/exp 5. 1 OR 2 6. 5 AND 3 7. 6 NOT 4 |
| **The world health organization international clinical trials platform search portal (WHO-ICTRP)**  **(2164 records found on January 29^th^, 2021)** | “angiotensin converting enzyme inhibitor” OR acei OR “ace inhibitor” OR captopril OR cilazapril OR enalapril OR fosinopril OR lisinopril OR perindopril OR quinapril OR ramipril OR teprotide OR benazepril OR trandolapril |
| **Clinicalgovtrials**  **(8 records found on January 29^th^, 2021)** | Condition："angiotensin converting enzyme inhibitor" OR acei OR "ace inhibitor" OR captopril OR cilazapril OR enalapril OR fosinopril OR lisinopril OR perindopril OR quinapril OR ramipril OR teprotide OR benazepril OR trandolapril  Other terms：pneumonia OR pneumonitis |

**Supplementary Table S3: List of studies included in the review and type of study**

|  | Information of the included articles | Type of study |
| --- | --- | --- |
| 1 | Ohkubo T, Chapman N, Neal B, et al. Effects of an angiotensin-converting enzyme inhibitor-based regimen on pneumonia risk. Am J Respir Crit Care Med. 2004;169(9):1041-1045. doi:10.1164/rccm.200309-1219OC | RCT |
| 2 | Lee JS, Chui PY, Ma HM, et al. Does Low Dose Angiotensin Converting Enzyme Inhibitor Prevent Pneumonia in Older People With Neurologic Dysphagia--A Randomized Placebo-Controlled Trial. J Am Med Dir Assoc. 2015;16(8):702-707. doi:10.1016/j.jamda.2015.05.009 | RCT |
| 3 | Widimský J, Kremer HJ, Jerie P, Uhlír O. Czech and Slovak spirapril intervention study (CASSIS). A randomized, placebo and active-controlled, double-blind multicentre trial in patients with congestive heart failure. Eur J Clin Pharmacol. 1995;49(1-2):95-102. doi:10.1007/BF00192366 | RCT |
| 4 | Willenheimer R, Helmers C, Pantev E, et al. Safety and efficacy of valsartan versus enalapril in heart failure patients. Int J Cardiol. 2002;85(2-3):261-270. doi:10.1016/s0167-5273(02)00154-7 | RCT |
| 5 | Randomised placebo-controlled trial of effect of ramipril on decline in glomerular filtration rate and risk of terminal renal failure in proteinuric, non-diabetic nephropathy. The GISEN Group (Gruppo Italiano di Studi Epidemiologici in Nefrologia). Lancet. 1997;349(9069):1857-1863. | RCT |
| 6 | Køber L, Torp-Pedersen C, Carlsen JE, et al. A clinical trial of the angiotensin-converting-enzyme inhibitor trandolapril in patients with left ventricular dysfunction after myocardial infarction. Trandolapril Cardiac Evaluation (TRACE) Study Group. N Engl J Med. 1995;333(25):1670-1676. doi:10.1056/NEJM199512213332503 | RCT |
| 7 | Hou FF, Zhang X, Zhang GH, et al. Efficacy and safety of benazepril for advanced chronic renal insufficiency. N Engl J Med. 2006;354(2):131-140. doi:10.1056/NEJMoa053107 | RCT |
| 8 | Davis TME, Davis WA. Influence of Renin-Angiotensin System Inhibitors on Lower-Respiratory Tract Infections in Type 2 Diabetes: The Fremantle Diabetes Study Phase II. Diabetes Care. 2020;43(9):2113-2120. doi:10.2337/dc20-0895 | Cohort Study |
| 9 | Kumazawa R, Jo T, Matsui H, Fushimi K, Yasunaga H. Association between Angiotensin-Converting Enzyme Inhibitors and Post-Stroke Aspiration Pneumonia. J Stroke Cerebrovasc Dis. 2019;28(12):104444. doi:10.1016/j.jstrokecerebrovasdis.2019.104444 | Cohort Study |
| 10 | Lai CC, Wang YH, Wang CY, Wang HC, Yu CJ, Chen L. Comparative effects of angiotensin-converting enzyme inhibitors and angiotensin II receptor blockers on the risk of pneumonia and severe exacerbations in patients with COPD. Int J Chron Obstruct Pulmon Dis. 2018;13:867-874. doi:10.2147/COPD.S158634 | Cohort Study |
| 11 | Soto M, Bang S, McCombs J, Rodgers K. Incidence of tuberculosis and pneumonia in a newly diagnosed type2 diabetic population and the impact of RAS therapy. Value in Health. 2017;20(5):A166. | Cohort Study |
| 12 | Soto M, Bang S, McCombs J, Rodgers KE. Renin Angiotensin system-modifying therapies are associated with improved pulmonary health. Clin Diabetes Endocrinol. 2017;3:6. doi:10.1186/s40842-017-0044-1 | Cohort Study |
| 13 | Bang S, Soto M, Rodgers K, McCombs J. The impact of RAS-modifying medications on the risk of pulmonary infections in hypertensive patients with type 1 diabetes. Value in Health. 2017; 20(5):A165. | Cohort Study |
| 14 | Bang S, Soto M, McCombs J, Rodgers K. The Effect of Ras-Modifying Medications on Pulmonary Complications in Patients with Hypertension. Value in Health; 2016;19(7):A642. | Cohort Study |
| 15 | Bang S, Soto M, Rodgers K, McCombs J. The effect of ras-modifying medications on pulmonary health in patients with type 2 diabetes mellitus. Value in Health; 2015;18(3):A57. | Cohort Study |
| 16 | Ishifuji T, Sando E, Kaneko N, et al. Recurrent pneumonia among Japanese adults: disease burden and risk factors. BMC Pulm Med. 2017;17(1):12. doi:10.1186/s12890-016-0359-1 | Cohort Study |
| 17 | Chang CH, Lin JW, Ruan SY, et al. Comparing individual angiotensin-converting enzyme inhibitors with losartan in the risk of hospitalization for pneumonia and related mortality: a nationwide cohort study. J Hypertens. 2015;33(3):634-643. doi:10.1097/HJH.0000000000000438 | Cohort Study |
| 18 | Wang HC, Lin CC, Lau CI, Chang A, Kao CH. Angiotensin-converting enzyme inhibitors and bacterial pneumonia in patients with Parkinson disease. Mov Disord. 2015;30(4):593-596. doi:10.1002/mds.26136 | Cohort Study |
| 19 | Shah S, McArthur E, Farag A, et al. Risk of hospitalization for community acquired pneumonia with renin-angiotensin blockade in elderly patients: a population-based study. PLoS One. 2014;9(10):e110165. doi:10.1371/journal.pone.0110165 | Cohort Study |
| 20 | Alsumrain M, Melillo N, Debari VA, et al. Predictors and outcomes of pneumonia in patients with spontaneous intracerebral hemorrhage. J Intensive Care Med. 2013;28(2):118-123. doi:10.1177/0885066612437512 | Cohort Study |
| 21 | Sato Y, Nakano K, Sato Y, Toshiyasu T, Kawabata K, Takahashi S. Angiotensin-converting enzyme (ACE) inhibitors could prevent aspiration pneumonia in head and neck cancer patients treated with concurrent chemoradiotherapy (CCRT). Eur. J. Cancer 2013; 49: S767-S768. | Cohort Study |
| 22 | Cuifang, S. The Effects of Angiotensin-Converting-Enzyme Inhibitor on Pneumonia in Older Stroke Patients. Circulation, 2010; 122(2): E348. | Cohort Study |
| 23 | Harada J, Sekizawa K. Angiotensin-converting enzyme inhibitors and pneumonia in elderly patients with intracerebral hemorrhage. J Am Geriatr Soc. 2006;54(1):175-176. doi:10.1111/j.1532-5415.2005.00575_8.x | Cohort Study |
| 24 | Arai T, Sekizawa K, Ohrui T, et al. ACE inhibitors and protection against pneumonia in elderly patients with stroke. Neurology. 2005;64(3):573-574. doi:10.1212/01.WNL.0000150897.14961.0F | Cohort Study |
| 25 | Arai T, Yasuda Y, Takaya T, et al. ACE inhibitors and reduction of the risk of pneumonia in elderly people. Am J Hypertens. 2000;13(9):1050-1051. doi:10.1016/s0895-7061(00)00301-0 | Cohort Study |
| 26 | Arai T, Yasuda Y, Takaya T, et al. Angiotensin-converting enzyme inhibitors, angiotensin-II receptor antagonists, and pneumonia in elderly hypertensive patients with stroke. Chest. 2001;119(2):660-661. doi:10.1378/chest.119.2.660 | Cohort Study |
| 27 | Shibuya S, Murahashi M, Inoue M, Jimi T, Wakayama Y. ACE inhibitors and its usefulness in the prevention of aspiration pneumonia in chronic cerebrovascular disease patients with asymptomatic swallowing dysfunction. Rinsho Shinkeigaku. 2002;42(3):240-242. | Cohort Study |
| 28 | Teramoto S, Ouchi Y. ACE inhibitors and prevention of aspiration pneumonia in elderly hypertensives. Lancet. 1999;353(9155):843. doi:10.1016/S0140-6736(05)76506-5 | Cohort Study |
| 29 | Sekizawa K, Matsui T, Nakagawa T, Nakayama K, Sasaki H. ACE inhibitors and pneumonia. Lancet. 1998;352(9133):1069. doi:10.1016/S0140-6736(05)60114-6 | Cohort Study |
| 30 | Chhibber A, Alexander D, Nickman N, Biskupiak J, Munger M. PIH3 Angiotensin I converting enzyme inhibitors reduce risk of pneumonia in hospitalized elderly patients. Value in Health, 2020; 23, S151. | Case control study |
| 31 | Shah R, Mousa O, Paredes A, Vaidya G, Rawlins S. Factors Increasing the Risk of Pneumonia Among Proton Pump Inhibitor Users. Am. J. Gastroenterol.2014; 109: S634. | Case control study |
| 32 | de Groot MC, Klungel OH, Leufkens HG, van Dijk L, Grobbee DE, van de Garde EM. Sources of heterogeneity in case-control studies on associations between statins, ACE-inhibitors, and proton pump inhibitors and risk of pneumonia. Eur J Epidemiol. 2014;29(10):767-775. doi:10.1007/s10654-014-9941-0 | Case control study |
| 33 | Dublin S, Walker RL, Jackson ML, Nelson JC, Weiss NS, Jackson LA. Angiotensin-converting enzyme inhibitor use and pneumonia risk in community-dwelling older adults: results from a population-based case-control study. Pharmacoepidemiol Drug Saf. 2012;21(11):1173-1182. doi:10.1002/pds.3340 | Case control study |
| 34 | Pope HE, Anger B, Malmstrom TK, Feen ES. Evaluation of ace inhibitor use and the prevention of pneumonia in stroke patients. Neurocrit. Care, 2012; 17: S40. | Case control study |
| 35 | Vilanova MB, Falguera M, Pena M, et al. Obesity and Metabolic Syndrome as Risk Factors for Community-Acquired Pneumonia. Clin. Microbiol. Infect. 2012: 136–137. | Case control study |
| 36 | Mukamal KJ, Ghimire S, Pandey R, O'Meara ES, Gautam S. Antihypertensive medications and risk of community-acquired pneumonia. J Hypertens. 2010;28(2):401-405. doi:10.1097/HJH.0b013e3283330948 | Case control study |
| 37 | Myles PR, Hubbard RB, McKeever TM, Pogson Z, Smith CJ, Gibson JE. Risk of community-acquired pneumonia and the use of statins, ace inhibitors and gastric acid suppressants: a population-based case-control study. Pharmacoepidemiol Drug Saf. 2009;18(4):269-275. doi:10.1002/pds.1715 | Case control study |
| 38 | Marciniak C, Korutz AW, Lin E, Roth E, Welty L, Lovell L. Examination of selected clinical factors and medication use as risk factors for pneumonia during stroke rehabilitation: a case-control study. Am J Phys Med Rehabil. 2009;88(1):30-38. doi:10.1097/PHM.0b013e3181909b73 | Case control study |
| 39 | van de Garde EM, Souverein PC, van den Bosch JM, Deneer VH, Leufkens HG. Angiotensin-converting enzyme inhibitor use and pneumonia risk in a general population. Eur Respir J. 2006;27(6):1217-1222. doi:10.1183/09031936.06.00110005 | Case control study |
| 40 | van de Garde EM, Souverein PC, Hak E, Deneer VH, van den Bosch JM, Leufkens HG. Angiotensin-converting enzyme inhibitor use and protection against pneumonia in patients with diabetes. J Hypertens. 2007;25(1):235-239. doi:10.1097/HJH.0b013e328010520a | Case control study |
| 41 | Etminan M, Zhang B, Fitzgerald M, Brophy JM. Do angiotensin-converting enzyme inhibitors or angiotensin II receptor blockers decrease the risk of hospitalization secondary to community-acquired pneumonia? A nested case-control study. Pharmacotherapy. 2006;26(4):479-482. doi:10.1592/phco.26.4.479 | Case control study |
| 42 | Takahashi T, Morimoto S, Okaishi K, et al. Reduction of pneumonia risk by an angiotensin I-converting enzyme inhibitor in elderly Japanese inpatients according to insertion/deletion polymorphism of the angiotensin I-converting enzyme gene. Am J Hypertens. 2005;18(10):1353-1359. doi:10.1016/j.amjhyper.2005.04.020 | Case control study |
| 43 | Ohse H, Horiguchi H, Saito T, Nagata H. ACE inhibitors and pneumonia in stroke patients. Japanese J. Chest Dis. 2004;63(8):776–781. | Case control study |
| 44 | El Solh AA, Brewer T, Okada M, Bashir O, Gough M. Indicators of recurrent hospitalization for pneumonia in the elderly. J Am Geriatr Soc. 2004;52(12):2010-2015. doi:10.1111/j.1532-5415.2004.52556.x | Case control study |
| 45 | Okaishi K, Morimoto S, Fukuo K, et al. Reduction of risk of pneumonia associated with use of angiotensin I converting enzyme inhibitors in elderly inpatients. Am J Hypertens. 1999;12(8 Pt 1):778-783. doi:10.1016/s0895-7061(99)00035-7 | Case control study |

**Supplementary Table S4: List of studies excluded from the review and reasons for exclusion**

|  | Information of the excluded articles | Reason for exclusion |
| --- | --- | --- |
| 1 | Kang JH, Kao LT, Lin HC, Wang TJ, Yang TY. Do outpatient statins and ACEIs/ARBs have synergistic effects in reducing the risk of pneumonia? A population-based case-control study. PLoS One. 2018;13(6):e0199981. doi:10.1371/journal.pone.0199981 | Wrong intervention or comparison |
| 2 | Kim J, Lee JK, Heo EY, Chung HS, Kim DK. The association of renin-angiotensin system blockades and pneumonia requiring admission in patients with COPD. Int J Chron Obstruct Pulmon Dis. 2016;11:2159-2166. doi:10.2147/COPD.S104097 | Wrong intervention or comparison |
| 3 | Kim J, Yim H, Park S et al. The Association of Angiotensin Converting Enzyme Inhibitors (ACEI)/Angiotensin II Receptor Antagonists (ARB) and Pneumonia Requiring Admission in Patients with Chronic Obstructive Pulmonary Disease (COPD) .Am. J. Respir. Crit. Care Med., 2014; 189: A1124. | Wrong intervention or comparison |
| 4 | Nakashima T, Hattori N, Okimoto M, Yanagida J, Kohno N. Nicergoline improves dysphagia by upregulating substance P in the elderly. Medicine (Baltimore). 2011;90(4):279-283. doi:10.1097/MD.0b013e318224413b | Wrong intervention or comparison |
| 5 | Arai T, Yasuda Y, Takaya T, et al. Angiotensin-converting enzyme inhibitors, angiotensin II receptor antagonists, and symptomless dysphagia. Chest. 2000;117(6):1819-1820. doi:10.1378/chest.117.6.1819 | Wrong intervention or comparison |
| 6 | Eficacia de Sevikar® comparado con la combinación de Perindoprilo/Amlodipino sobre la Presión Arterial Central, en Pacientes con Hipertensión moderada a severa - SEVITENSION​ (online). Available at: https://www.clinicaltrialsregister.eu/ctr-search/search?query=eudract_number:2009-012966-30. Accessed April 20, 2021. | Wrong intervention or comparison |
| 7 | Fominaya CE, Brown J, Doloresco F, Monte SV. Respiratory Outcomes Associated with Inhibition of the Reninangiotensin-Aldosterone System among Patients with Chronic Obstructive Pulmonary Disease. Pharmacotherapy. 2010;30(10):449e. | Wrong intervention or comparison |
| 8 | Henry C, Zaizafoun M, Stock E, Ghamande S, Arroliga AC, White HD. Impact of angiotensin-converting enzyme inhibitors and statins on viral pneumonia. Proc (Bayl Univ Med Cent). 2018;31(4):419-423. doi:10.1080/08998280.2018.1499293 | Wrong population |
| 9 | Zaizafoun M, Henry C, White HD, et al. Impact of Angiotensin Converting Enzyme (ACE) Inhibitors and Statins on Outcomes in Viral Pneumonia.” Am. J. Respir. Crit. Care Med. 2015;191:A1761. | Wrong population |
| 10 | Christiansen CF, Heide-Jørgensen U, Rasmussen TB, et al. Renin-Angiotensin System Blockers and Adverse Outcomes of Influenza and Pneumonia: A Danish Cohort Study. J Am Heart Assoc. 2020;9(19):e017297. doi:10.1161/JAHA.120.017297 | Wrong population |
| 11 | Sio TT, Atherton PJ, Pederson LD, et al. Daily Lisinopril vs Placebo for Prevention of Chemoradiation-Induced Pulmonary Distress in Patients With Lung Cancer (Alliance MC1221): A Pilot Double-Blind Randomized Trial. Int J Radiat Oncol Biol Phys. 2019;103(3):686-696. doi:10.1016/j.ijrobp.2018.10.035 | Wrong outcome |
| 12 | Chang C, Song X, Sainski-Nguyen A et al. Hospitalization in patients with heart failure and reduced ejection fraction and diabetes treated with sacubitril/valsartan versus angiotensin-converting enzyme inhibitor or angiotensin receptor blocker. J Manag Care Spec Pharm [Internet]. 2018; 24(10A):S63. | Wrong outcome |
| 13 | Albert Wu. The association of cardioprotective medications with pneumonia-related outcomes. J. Am. Geriatr. Soc., 2012; 60: S146–7. | Wrong outcome |
| 14 | Yoshimura K, Ozawa Y, Matsui T, Yokomura K, Suda T, Chida K. Retrospective Study for Exploring Preventive Effect of Ace Inhibitors on Acute Exacerbation of COPD. Am. J. Respir. Crit. Care Med. 2013;187:A2426. | Wrong outcome |
| 15 | Mortensen EM, Nakashima B, Cornell J, et al. Population-based study of statins, angiotensin II receptor blockers, and angiotensin-converting enzyme inhibitors on pneumonia-related outcomes. Clin Infect Dis. 2012;55(11):1466-1473. doi:10.1093/cid/cis733 | Wrong outcome |
| 16 | Shimizu T, Fujioka S, Otonashi H, Kondo M, Sekizawa K. ACE inhibitor and swallowing difficulties in stroke. A preliminary study. J Neurol. 2008;255(2):288-289. doi:10.1007/s00415-006-0157-4 | Wrong outcome |
| 17 | Nakayama K, Sekizawa K, Sasaki H. ACE inhibitor and swallowing reflex. Chest. 1998;113(5):1425. doi:10.1378/chest.113.5.1425 | Wrong outcome |
| 18 | Chen N. Effectiveness Study on Fosinopril and/or Losartan in Patients With Chronic Kidney Disease Stage 3 (FLIP)(online). Available at: https://clinicaltrials.gov/ct2/show/NCT00565396. Accessed April 20, 2021. | Wrong outcome |
| 19 | Heart Outcomes Prevention Evaluation Study Investigators, Yusuf S, Sleight P, et al. Effects of an angiotensin-converting-enzyme inhibitor, ramipril, on cardiovascular events in high-risk patients [published correction appears in 2000 May 4;342(18):1376] [published correction appears in N Engl J Med 2000 Mar 9;342(10):748]. N Engl J Med. 2000;342(3):145-153. doi:10.1056/NEJM200001203420301 | Wrong outcome |
| 20 | Kanda A, Ebihara S, Yasuda H, Takashi O, Sasaki T, Sasaki H. A combinatorial therapy for pneumonia in elderly people. J Am Geriatr Soc. 2004;52(5):846-847. doi:10.1111/j.1532-5415.2004.52230_5.x | Wrong outcome |
| 21 | ONTARGET Investigators, Yusuf S, Teo KK, et al. Telmisartan, ramipril, or both in patients at high risk for vascular events. N Engl J Med. 2008;358(15):1547-1559. doi:10.1056/NEJMoa0801317 | Wrong outcome |
| 22 | Mortensen EM, Restrepo MI, Anzueto A, Pugh J. The impact of prior outpatient ACE inhibitor use on 30-day mortality for patients hospitalized with community-acquired pneumonia. BMC Pulm Med. 2005;5:12. doi:10.1186/1471-2466-5-12 | Wrong outcome |
| 23 | Mortensen EM, Pugh MJ, Copeland LA, et al. Impact of statins and angiotensin-converting enzyme inhibitors on mortality of subjects hospitalised with pneumonia. Eur Respir J. 2008;31(3):611-617. doi:10.1183/09031936.00162006 | Wrong outcome |
| 24 | Chalmers JD, Singanayagam A, Murray MP, Hill AT. Prior statin use is associated with improved outcomes in community-acquired pneumonia. Am J Med. 2008;121(11):1002-1007.e1. doi:10.1016/j.amjmed.2008.06.030 | Wrong outcome |
| 25 | Myles PR, Hubbard RB, Gibson JE, Pogson Z, Smith CJ, McKeever TM. The impact of statins, ACE inhibitors and gastric acid suppressants on pneumonia mortality in a UK general practice population cohort. Pharmacoepidemiol Drug Saf. 2009;18(8):697-703. doi:10.1002/pds.1769 | Wrong outcome |
| 26 | Maurer JR. Do statins and/or ACE inhibitors impact risk for or outcome of pneumonia in certain populations?. Curr Infect Dis Rep. 2008;10(3):213-214. doi:10.1007/s11908-008-0035-6 | Wrong  Outcome |
| 27 | Liu CL, Shau WY, Chang CH, Wu CS, Lai MS. Pneumonia risk and use of angiotensin-converting enzyme inhibitors and angiotensin II receptor blockers. J Epidemiol. 2013;23(5):344-350. doi:10.2188/jea.je20120112 | Wrong study design |
| 28 | Junker A. Less Pneumonia after Use of ACE Inhibitors: Cough Due to ACE Inhibitors with Protective Effect. Deutsche Apotheker Zeitung. 2012;152.35:36–37. | Wrong study design |
| 29 | ACE inhibitors may lower pneumonia risk. (2012, October). Harvard Health Letter / from Harvard Medical School. | Wrong study design |
| 30 | Berge E. Can angiotensin-converting enzyme inhibitors reduce the risk of pneumonia after stroke?. J Hypertens. 2012;30(11):2088-2089. doi:10.1097/HJH.0b013e328358bb10 | Wrong study design |
| 31 | Barnes RA. Pneumonia and ACE inhibitors--and cough. BMJ. 2012;345:e4566. doi:10.1136/bmj.e4566 | Wrong study design |
| 32 | Popular cardiac drug may prevent pneumonia. Harv Mens Health Watch. 2012;17(3):8. | Wrong study design |
| 33 | Matsumoto S, Shimodozono M, Toyama K, Kawahira K. Beneficial Effect of Perindopril in Post-Stroke Patients with Dysphagia. Dysphagia, 2012; 27(4):605. | Wrong study design |
| 34 | Liu CL, Shau WY, Wu CS, Lai MS. Angiotensin-converting enzyme inhibitor/angiotensin II receptor blockers and pneumonia risk among stroke patients. J Hypertens. 2012;30(11):2223-2229. doi:10.1097/HJH.0b013e328357a87a | Wrong study design |
| 35 | Liu CL, Shau WY, Wu CS et al. Angiotensin converting enzyme inhibitor (ACEI)/ angiotensin ii receptor blockers (ARB) and pneumonia risk among stroke patients. Pharmacoepidemiol Drug Saf. 2011;20:S80. | Wrong study design |
| 36 | Mascitelli L, Pezzetta F, Goldstein MR. Inhibition of the renin-angiotensin system in severe COPD. Eur Respir J. 2008;32(4):1130-1131. doi:10.1183/09031936.00082308 | Wrong study design |
| 37 | Teramoto S, Yamamoto H, Yamaguchi Y, et al. ACE inhibitors prevent aspiration pneumonia in Asian, but not Caucasian, elderly patients with stroke. Eur Respir J. 2007;29(1):218-220. doi:10.1183/09031936.00115106 | Wrong study design |
| 38 | Serafin-Bromblik J, Bartula M, Marcisz C. Inhibitory enzymu konwertujqcego angiotensyng a uklad oddechow [Angiotensin converting enzyme inhibitors and respiratory system]. Pol Merkur Lekarski. 2006;21(123):286-290. | Wrong study design |
| 39 | Sica DA, Brath L. Angiotensin-converting enzyme inhibition-emerging pulmonary issues relating to cough. Congest Heart Fail. 2006;12(4):223-226. doi:10.1111/j.1527-5299.2006.05746.x | Wrong study design |
| 40 | Sekizawa K. Inhibitors of angiotensin-converting enzyme and physical function in older women. Lancet. 2002;360(9339):1099-1100. doi:10.1016/S0140-6736(02)11172-X | Wrong study design |
| 41 | Arai T, Yasuda Y, Takaya T, et al. Technetium tin colloid test detecting symptomless dysphagia and ACE inhibitor prevented occurrence of aspiration pneumonia. Int J Mol Med. 2000;5(6):609-610. doi:10.3892/ijmm.5.6.609 | Wrong study design |
| 42 | Kaplan RC, Psaty BM. ACE-inhibitor therapy and nosocomial pneumonia. Am J Hypertens. 1999;12(11 Pt 1):1161-1162. doi:10.1016/s0895-7061(99)00166-1 | Wrong study design |
| 43 | Chatterjee SK. Role of ACE inhibitors on pneumonia. J Indian Med Assoc. 1999;97(6):242. | Wrong study design |
| 44 | Maeda K, Wakabayashi H, Shamoto H. Angiotensin-Converting Enzyme Inhibitor in Tube-Fed Patients With Stroke History. J Am Med Dir Assoc. 2015;16(10):896-897. doi:10.1016/j.jamda.2015.07.006 | Wrong study design |
| 45 | Ishifuji T, Sando E, Kaneko N, et al. Medications associated with the incidence of recurrent pneumonia in Japanese elderly population. Eur Respir J [Internet], 2015;46: S59. | Abstracts of other included studies |
| 46 | Ruan SY, Chang CH, Lee YC, et al. Comparing Individual Angiotensin-Converting Enzyme Inhibitors with Angiotensin Receptor Blockers on the Risk of Pneumonia: A Nationwide Cohort Study. Pharmacoepidemiol Drug Saf. 2014;23:461–462. | Abstracts of other included studies |
| 47 | Alsumrain M, Melillo N, Moussavi M, Kirmani J. Predictors and outcome of pneumonia in patients with spontaneous intracerebral hemorrhage. Am J Respir Crit Care Med [Internet], 2010;181(1). | Abstracts of other included studies |
| 48 | de Groot MC, Klungel OH, Leufkens HG, Leufkens HGM, et al. Multiple Database Approach for Study of Associations between Frequently Used Drugs and Community-Acquired Pneumonia. Pharmacoepidemiol Drug Saf, 2013; 22: 289. | Abstracts of other included studies |
| 49 | Sascha Dublin, Rod L Walker, Michael L Jackson et al. Use of Angiotensin-Converting Enzyme Inhibitors Is Not Associated with Decreased Pneumonia Risk.” Pharmacoepidemiol Drug Saf , 2011: S83–S84. | Abstracts of other included studies |
| 50 | Arai T, Yasuda Y, Toshima S, Yoshimi N, Kashiki Y. ACE inhibitors and pneumonia in elderly people. Lancet. 1998;352(9144):1937-1938. doi:10.1016/S0140-6736(05)60437-0 | Same RCTs of other included studies |
| 51 | PROGRESS Collaborative Group. Randomised trial of a perindopril-based blood-pressure-lowering regimen among 6,105 individuals with previous stroke or transient ischaemic attack [published correction appears in Lancet 2001 Nov 3;358(9292):1556] [published correction appears in Lancet 2002 Jun 15;359(9323):2120]. Lancet. 2001;358(9287):1033-1041. doi:10.1016/S0140-6736(01)06178-5 | Same RCTs of other included studies |
| 52 | Mori T. Effect of perindopril on prevention of aspiration pneumonia following severe intracerebral hemorrhage (EPOCH) study(online). Available at: https://upload.umin.ac.jp/cgi-open-bin/ctr_e/ctr_view.cgi?recptno=R000009625. Accessed April 20, 2021. | Protocols without results |

| **Supplementary Table S5: Main characteristics of randomized controlled trials included in the review** | | | | | | | | |
| --- | --- | --- | --- | --- | --- | --- | --- | --- |
| Study | Location | Mean follow-up length (years) | Patients | Comparison | No (total) | Mean age (SD) | Primary Outcome in the original article | Outcome abstracted |
| Lee 2015 | Hong Kong  Multicenter | 0.5 | Tube fed patients aged ≥ 60 yo with history of recent hospitalization | Placebo | 93 | 83.9  (6.2) | Incidence rate of pneumonia within 26-week trial period | Pneumonia |
| Hou 2006 | China  Single center | 3.4 | Patients with nondiabetic chronic kidney disease | Placebo | 224 | 44.7  (15.5) | Time to the first event in the composite end point of a doubling o f the serum creatinine level, end-stage renal disease, or death. | Pneumonia as cause of mortality |
| Ohkubo 2004 | Worldwide  Multicenter | 3.9 | Patients with history of stroke or TIA within the past 5 years | Placebo | 6105 | 64  (10) | Risk of pneumonia | Fatal or non-fatal pneumonia |
| Willenheimer 2002 | Sweden  NR | 0.23 | Patients with stable heart failure | Valsartan | 141 | 68 (NR) | Exercise capacity measured as the distance walked during a 6 min-walk test | Death due to pneumonia |
| The GISEN Group 1997 | Italy  Multicenter | 1.3 | Patients with chronic nephropathy and persistent proteinuria | Placebo | 166 | 49.3  (13.6) | Rate of GFR decline | Drug withdrawal due to bronchopneumonia |
| Widimský 1995 | Czech and Slovak  Multicenter | 0.2 | Patients with congestive heart failure due to coronary heart disease or dilated cardiomyopathy | Placebo | 248 | 57.5  (10) | Change in exercise duration from baseline to endpoint, measured by programmable, electronically braked bicycle | Serious pneumonia |
| Køber 1995 | Denmark  Multicenter | 2.17 | Patients with myocardial infarction within 7 days | Placebo | 1749 | 69  (NR) | Death from any cause | Pneumonia |

Abbreviations: TIA: Transient ischemic attack, GFR: Glomerular filtration rate, SD: Standard deviation, NR: Not reported, yo: year-old

| **Supplementary Table S6: Main characteristics of cohort studies included in the review** | | | | | | | | | | | |
| --- | --- | --- | --- | --- | --- | --- | --- | --- | --- | --- | --- |
| Study | Location study  design | Study  length (years) | Data source period of study | Patient | Comparison | No  (total) | Mean age  (SD) | Outcome measures | Ascertainment Drug use | Ascertainment Outcomes | Outcome adjustments for confounders |
| Davis 2020 | Australia retrospective | 6.4 | The Hospital Morbidity Data Collection and community-based cohort at the city of Fremantle in the state of Western Australia  2008 to 2016 | Patients in the catchment area with a clinician-verified diagnosis of DM | ACE-I  v Control* | 1482 | 65.8  (11.6) | Hospitalizations for the main bacterial lung parenchymal infections pneumonia | Face-to-face interview at entry, and verification of medication and prescription at each visit. | ICD-10 codes | Age, DM, CKD, CHF, COPD, Asthma, Stroke, Antacid |
| Kumazawa  2019 | Japan retrospective | 0.25 | Diagnosis Procedure Combination database (a nationwide inpatient database)  July 1, 2010 to March 31, 2017 | Patients hospitalized for stroke and developed pneumonia during hospitalization | ACE-I  v ARB | 11578 | 81  (NR) | 90-day readmission for post-stroke pneumonia | Medication record at discharge extracted from the database | ICD-10 codes | Age, DM, History of pneumonia, Gender, Stroke, Antacid, Oral corticosteroid, Immunosuppressants, Smoking |
| Lai 2018 | Taiwan retrospective | 5.9 | The Taiwan National Health Insurance Research Database 2000 to December 31, 2011 | COPD patients ≥ 40 years who received prescriptions for an ACE-I or ARB | ACE-I  v ARB | 12452 | 67.3 (9.9) | Pneumonia | Prescription record from the database | ICD-9 codes | Age, DM, CKD, CHF, Gender, COPD, Stroke, Antacid |
| Soto 2017-2 | the US retrospective | NR | Claims data from a US commercial NR | Patients with type2 DM taking hypertensive medications | ACE-I  v CCB or Diuretics | NR | NR | Pneumonia | Medication record from the database | NR | NR |
| Soto 2017-1 | the US retrospective | NR | The Humana dataset, a United States-based insurance claims dataset January 1, 2007 to June 30, 2013 | Patients with HTN treated with hypertensive medications | ACE-I  v CCB or Diuretics | 190699 | 63.5 (13.7) | The first pulmonary or lung complication diagnosis | Medication record from the database | ICD-9 codes | Age, DM, Gender, COPD, Asthma, Flu vaccination |
| Bang 2017 | the US retrospective | NR | Claims data from a US commercial insurance company NR | Patients with HTN and type1 DM treated with hypertensive medications | ACE-I v CCB or Diuretics | NR | NR | Pneumonia | Medication record from the database | NR | NR |
| Bang 2016 | the US retrospective | NR | Claims data from a US commercial insurance company NR | Patients with HTN and type1 DM treated with hypertensive medications | ACE-I v CCB or Diuretics | 97182 | NR | Pneumonia and influenza | Medication record from the database | ICD-9 codes | DM |
| Bang 2015 | the US retrospective | 4.5 | Claims data from a US commercial insurance company NR | Patients with type2 DM taking hypertensive medications | ACE-I v CCB or Diuretics | 171857 | NR | Pneumonia and influenza | Medication record from the database | ICD-9 codes | NR |
| Ishifuji 2017 | Japan prospective | 1.3 | Hospital based cohort at Kameda General Hospital February 1, 2012 to January 31, 2013 | Patients aged ≥ 15 yo with pneumonia | ACE-I v Control | 841 | 73  (Median) | Recurrent pneumonia | Medication information prospectively collected by hospital physicians and a research clinician at actual visit. | Independent review of medical records and patients’ thoracic diagnostic images and results | Age, DM, CHF, History of pneumonia, Gender, COPD, Asthma, Antacid, Oral corticosteroid, Immunosuppressants |
| Chang 2015 | Taiwan retrospective | 0.21 | The Taiwan National Health Insurance Research Database January 1, 2004 to December 31, 2010 | Patients ≥ 20 yo who initiated ACE-I | ACE-I  v Losartan | 1339169 | 60.0 (14.2) | Hospitalization for pneumonia | Medication record from the outpatient pharmacy prescription database | ICD-9 codes | Age, CKD, CHF, Gender, COPD, Asthma, Stroke, Oral corticosteroid |
| Wang 2015 | Taiwan retrospective | 3.7 | The Longitudinal Health Insurance Database, which is a component of the National Health Insurance Research Database 2001 to December 31,2010 | Patients ≥ 50 yo with PD and HTN | ACE-I v Control | 2310 | 73.6 (9.8) | Pneumonia | Anatomical Therapeutic Chemical (ATC) Code from the database | ICD-9 codes | Age, DM, History of pneumonia, Gender, COPD, Asthma, Stroke |
| Shah 2014 | Canada retrospective | 0.25 | Health administrative data from Ontario, Canada June 1, 2003 to December 31, 2011 | Patients ≥ 65 y0prescribed hypertensive medications | ACE-I v Control | 121015 | 73.3 (6.1) | Hospitalization for pneumonia | Prescription record from Ontario Drug Benefits (ODB) database | ICD-10 codes | Age, DM, CKD, CHF, History of pneumonia, gender, COPD, Asthma, Stroke |
| Alsumrain 2013 | the US retrospective | NR | New Jersey Neuroscience Institute, John F Kennedy Hospital in Edison, New Jersey NR | Patients with the diagnosis of intracerebral hemorrhage who were admitted within 24 hours of onset of stroke | ACE-I v Control | 290 | 66.6 (16.2) | Pneumonia | Medication history collected by medical staff at admission | A constellation of suggestive clinical features and a demonstrable infiltrate by chest radiograph or other imaging technique | Stroke |
| Sato 2013 | Japan retrospective | 1.6 | Cancer Institute Hospital of Japanese Foundation of Cancer Research  November, 2005 to December, 2012 | Patients with non-metastatic head and neck cancer treated with CCRT | ACE-I v Other hypertensive medications | 73 | 59.6  (NR) | Aspiration pneumonia | NR | NR | NR |
| Cuifang 2010 | China prospective | NR | NR | Patients ≥ 60 yo with HTN and stroke | ACE-I v Control | 489 | NR | Pneumonia | Prescription record | NR | NR |
| Harada 2006 | Japan prospective | 2.0 | NR | Patients ≥ 65 yo with a history of stroke, but not bedridden | ACE-I v Control | 83 | 68 (18.2) | Pneumonia | NR | A new pulmonary infiltrate seen on a chest radiograph and one of the following features: cough, temperature greater than 37.8°C, or subjective dyspnea | NR |
| Arai 2005 | Japan prospective | 2.9 | NR April, 1999 to February, 2002 | Patients with stroke | ACE-I v Control | 590 | 75.3 (1.1) | Pneumonia | NR | A new pulmonary infiltrate seen on a chest radiograph and one of the following features: cough, temperature greater than 37.8°C, or subjective dyspnea | Stroke |
| Shibuya 2002 | Japan prospective | 3.0 | NR | Patients with stroke who had abnormality of their swallowing function | ACE-I v Control | 85 | NR | Pneumonia | NR | Questionnaire, x-ray, sputum culture | Stroke |
| Arai 2001 | Japan prospective | 2.0 | NR  January, 1998 to May, 2000 | Elderly patients with HTN and stroke | ACE-I v ARB | 404 | NR | Pneumonia | NR | NR | Stroke |
| Arai 2000 | Japan prospective | 4.0 | NR  January, 1995 to December, 1999 | Patients ≥ 65 yo treated with hypertensive medications | ACE-I v CCB | 879 | NR | Pneumonia | NR | NR | NR |
| Teramoto 1999 | Japan retrospective | 3.0 | NR  1995 to 1998 | Outpatients with HTN | ACE-I v CCB | 498 | 63.1 (7.0) | Pneumonia | NR | NR | NR |
| Sekizawa 1998 | Japan prospective | 2.0 | NR  March, 1996 to 1998 | Patients with stroke treated with hypertensive medications | Imidapril, enalapril, captopril, v CCB or β blocker | 440 | 76.7 (7.7) | Pneumonia | NR | Diagnosis of pneumonia was made by two radiologists who were not involved in the studies | Stroke |

Abbreviations: ACE-I: Angiotensin converting enzyme, ARB: Angiotensin 2 receptor blocker, HTN: Hypertension, DM: Diabetes Meletus, CKD: Chronic kidney disease, COPD: Chronic obstructive pulmonary disease, CHF: Congestive heart failure, SD: Standard deviation, NR: Not reported, yo: year-old, PD: Parkinson disease, CCRT: Concurrent chemoradiotherapy, ICD: International classification of diseases

*Inquired result (Originally intervention was ACE-I/ARB and outcome was Pneumonia/Flu in the study)

| **Supplementary Table S7: Main characteristics of case-control studies included in the review** | | | | | | | | | | | | |
| --- | --- | --- | --- | --- | --- | --- | --- | --- | --- | --- | --- | --- |
| Study | Location, study design | Data length (years) | Data source period of study | Patient | Control | Matching | No  (total) | Mean  Age  (SD) | Outcome | Ascertainment Drug use | Ascertainment Outcome | Outcome adjustments for confounders |
| Chhibber 2020 | the US  retrospective | 4 | University of Utah Data Warehouse January, 2012 to December, 2016 | Hospitalized patients aged ≥65 yo without ICD-diagnostic codes associated with dysphagia with a diagnosis of pneumonia | NR | Ratio 1:1  age, gender and Charlson’s Comorbidity Index | 3270 | NR | Pneumonia | Patient records were reviewed for the use of ACE-I at 3 months, 6 months and 9 months prior to the admission date | ICD codes | Age, DM, CKD, CHF, gender, COPD, Asthma, Stroke |
| Shah  2014-2 | the US retrospective | 1 | Electronic medical records of SUNY upstate university hospital  January, 2012 to December, 2012 | Patients ≥18 yo admitted to hospital with a primary admission diagnosis of pneumonia and on PPI at admission | Patients with a primary admission diagnosis of chest pain without evidence of pneumonia | NR | 221 | 58.1 (NR) | Admission for pneumonia | Medication record from electronic medical records | NR | NR |
| de Groot 2014 (LRGP) | Netherlands retrospective | 6 | Leidsche Rijn General Practitioner database(Dutch general practitioner databases) 2004 to 2010 | Patients having a record for pneumonia | Patients without a record for pneumonia in the 12 months prior to the index date | Ratio 1:2-5; sex and year of birth | 3145 | 45.6 (14.6) | Pneumonia | ATC Code from the database Drug use was defined as two or more prescriptions/dispenses in the 182 days before the index date | ICPC-2 codes | Age, DM, CKD, CHF, gender, COPD, Asthma, Stroke, Antacid, Oral corticosteroid, Flu vaccination |
| de Groot 2014 (AHC) | Netherlands retrospective | 6 | Almere Health Care group general practitioner database(Dutch general practitioner databases) 2004 to 2010 | Patients having a record for pneumonia | Patients without a record for pneumonia in the 12 months prior to the index date | Ratio 1:2-5; sex and year of birth | 16603 | 53.6 (17.6) | Pneumonia | ATC Code from the database drug use was defined as two or more prescriptions/dispenses in the 182 days before the index date | ICPC-2 codes | Age, DM, CKD, CHF, gender, COPD, Asthma, Stroke, Antacid, Oral corticosteroid, Flu vaccination |
| de Groot 2014 (NPCD) | Netherlands retrospective | 6 | Netherlands Primary Care Database (Dutch general practitioner databases) 2004 to 2010 | Patients having a record for pneumonia | Patients without a record for pneumonia in the 12 months prior to the index date | Ratio 1:2-5; sex and year of birth | 74357 | 60.7 (18.2) | Pneumonia | ATC Code from the database Drug use was defined as two or more prescriptions/dispenses in the 182 days before the index date | ICPC-2 codes | Age, DM, CHF, gender, COPD, Antacid, Oral corticosteroid, Flu vaccination |
| de Groot 2014 (PH) | Netherlands retrospective | 6 | PHARMO database 2004 to 2010 | Patients having a record for pneumonia | Patients without a record for pneumonia in the 12 months prior to the index date | Ratio 1:2-5; sex and year of birth | 62152 | 66 (17.1) | Pneumonia | ATC Code from the database Drug use was defined as two or more prescriptions/dispenses in the 182 days before the index date | ICD-9 codes | Age, DM, CHF, gender, COPD, Antacid, Oral corticosteroid, Flu vaccination |
| de Groot 2014 (ANT) | Netherlands retrospective | 6 | Medical record of two hospitals 2004 to 2010 | Patients having a record for pneumonia | Patients without a record for pneumonia in the 12 months prior to the index date | Ratio 1:2-5; sex and year of birth | 1837 | 61 (17) | Pneumonia | ATC Code from the database Drug use was defined as two or more prescriptions/dispenses in the 182 days before the index date | ICD-9 codes | Age, DM, CHF, Gender, COPD, Antacid, Oral corticosteroid |
| Dublin 2012 | the US retrospective | 3 | Group Health (GH), an integrated healthcare delivery system in Washington State, USA 2000 to 2003 | Community-dwelling GH members aged 65–94 yo having a record for pneumonia | Patients without a record for pneumonia during period of study after matching | Ratio 1:2 age, sex and calendar year. | 3061 | 77 (8.1) | Pneumonia | GH’s computerized pharmacy data Drug use was defined as filling ≥ 2 prescriptions during the 180 days prior to the case’s diagnosis date | ICD-9 codes and validated by emergency department visit, chest radiograph or other medical records. | Age, DM, CKD, CHF, Gender, COPD, Asthma, Oralcorticosteroid, Smoking, Flu vaccination, Pneumococcal vaccination |
| Pope 2012 | the US retrospective | 1.75 | Saint Louis University Hospital March 1, 2009 to November 30, 2011 | Patients admitted to hospital due to acute ischemic stroke, spontaneous intracerebral hemorrhage, or non-traumatic subarachnoid hemorrhage, with a record for pneumonia | Patients without a record for pneumonia during period of study after matching | Ratio 1:1.5 primary diagnosis, baseline demographics, history of prior stroke, DM, HTN, heart failure, and initial NIHSS scores | 237 | NR | Pneumonia | NR | ICD-9 codes or antibiotic treatment course for at least 7 days with a positive respiratory culture | Age, DM, CKD, CHF, Gender, COPD, Stroke |
| Vilanova  2012 | Spain retrospective | 1.25 | The Emergency Department of Arnau de Vilanova Hospital January, 2009 to March, 2010 | Patients ≥ 18 yo diagnosed as CAP in the Emergency Department | Patients randomly selected from a Primary Care | Ratio 1:1 age and sex | 328 | 68 (NR) | Pneumonia | NR | NR | Age, Diabetes, CKD, CHF, gender, Stroke, Smoking |
| Mukamal, 2010 | the US retrospective | 7.9 | Claims data for commercially insured individuals January, 2000 to November, 2007 | Patients with HTN with a record of pneumonia | Patients who did not have a preceding diagnosis of pneumonia as of the index date | Ratio 1:10  age, sex, US Census region of residence, insurance plan, subscriber status (insured individual, spouse, or dependent), and date of enrollment | 81000 | 58.2 (12.5) | Pneumonia | Claims in the 3 months preceding the index date, corresponding to the largest supply of medications paid on standard single claims | A primary hospitalization discharge diagnosis or two outpatient claims within a single month with ICD-9 codes of 480 – 486 | Age, DM, CKD, CHF, gender, COPD, Asthma, Stroke |
| Myles 2009 | the UK retrospective (population based cohort) | 1 | A comprehensive longitudinal database of primary care medical records called the health improvement network July 1, 2001 to July 1, 2002 | Patients aged ≥ 40 yo with a diagnosis of pneumonia | Patients without a record for pneumonia during period of study | Ratio 1:6 practice, sex and age at index date | 25883 | NR | Pneumonia | Prescription record of the database The most recent prescription was within 30 days before the pneumonia index date | ICD-9 codes | Age, DM, CKD, CHF, History of pneumonia, gender, COPD, Asthma, Stroke, Antacid. Oral corticosteroid, Smoking |
| Marciniak  2009 | the US retrospective | 4 | The Stroke Rehabilitation Registry Database at the Rehabilitation Institute of Chicago, IL. September 1999 to August 2003 | Patients ≥ 18 yo admitted for inpatient rehabilitation within 90 days of the onset of their stroke with a record of pneumonia | Patients who did not develop pneumonia during rehabilitation | Ratio1:1 Age, sex, type of stroke, NIH Stroke Scale score, side of stroke, depth of stroke | 72 | 66.3 (12.1) | Pneumonia | Review of the medical record | Review of medical record Clinical setting of fever, chills, muscle stiffness, chest pain, cough, shortness of breath, rapid heart rate, or difficulty breathing, with chest x-ray confirmation | Age, gender, Stroke, Antacid |
| van de Garde 2007 | the UK retrospective | 14 | General Practice Research Database June 1, 1987 to January 21, 2001 | Patients ≥ 18 yo with DM who had a first diagnosis of pneumonia | Patients in the database without a record for pneumonia | Ratio 1: upto 4 sex, age, general practice, index date | 20041 | 73 (11) | Pneumonia | Prescription record within a year before the index date based on the database | The Oxford Medical Information System and the Read codes | Age, DM, CHF, gender, COPD, Asthma, Stroke, Antacid, Oral corticosteroid, Smoking, Flu vaccination, Pneumococcal vaccination |
| van de Garde 2006 | Netherlands retrospective | 6 | The PHARMO record linkage system January 1, 1995 to December 31, 2000 | Patients ≥ 18 yo hospitalized with a primary discharge diagnosis of pneumonia and a secondary discharge diagnosis coupled with a primary pulmonary diagnosis | Patients without a record for pneumonia | Ratio 1: 1-4 sex, age | 4925 | 67 (0.51) | Pneumonia | ATC Code from the database between entry into the cohort and the index date | ICD-9 | Age, DM, CHF, gender, COPD, Asthma, Antacid, Oral corticosteroid |
| Etminan 2006 | Canada retrospective | 5 | Universal Quebec, Canada, administrative health databases 1996 to 2000 | Patients who underwent a coronary revascularization procedure and had a diagnosis of pneumonia | Patients without a record of pneumonia | Ratio 1:20 age, the calendar year of cohort entry | 34981 | 71 (8.2) | Pneumonia | Users were defined as those who had filled at least one prescription for an ACE-I within 7 days of the index date. | ICD-9 | Age, DM, CKD, CHF, History of pneumonia, Gender, COPD, Stroke |
| Takahashi 2005 | Japan  retrospective | 0.9 | Database from both an acute hospital and a long-term care facility  January to November in 1999 | Japanese patients ≥ 65 yo, with an admission period of 3 month who presented pneumonia | Patients without a record of pneumonia | Ratio 1:4 age, sex | 525 | 82.7 (8.2) | Pneumonia | Medication record from the hospital computerized pharmacy database | Definition of nosocomial pneumonia from CDC was applied; together with the stipulation that an abnormal finding on chest roentgenogram | Age, DM, CHF, Gender, COPD, Asthma, Oral corticosteroid |
| Ohse 2004 | Japan retrospective | 4 | Electrical database of National Hospital Science Ibaraki Prefectural University of Health Science  NR | Hospitalized patients due to stroke who had difficulty with swallowing function and had a pneumonia during hospitalization | Hospitalized patients due to stroke who had difficulty with swallowing function without pneumonia during hospitalization | NR | 95 | 62.7 (11.4) | Pneumonia | NR | NR | Stroke |
| El Solh 2004 | the US retrospective | 4.5 | An electronic database collected from three tertiary-care hospitals associated with the University at Buffalo March, 1999 to August 2003 | Patients ≥ 65 yo readmitted to the hospital with a clinical diagnosis of pneumonia over a period of 1 year from the first episode | Patients who had a non-recurrent pneumonia during the study period | Ratio 1:1 age, admission date, residence | 408 | 78.5 (8.1) | Readmission to the hospital with a clinical diagnosis of pneumonia | An electronic database | An electronic database Clinical manifestations or laboratory findings and the presence of a new pulmonary infiltrate on the chest radiograph | Age, History of pneumonia, COPD, Asthma, Smoking, Pneumococcal vaccination |
| Okaishi 1999 | Japan retrospective | 1 | Department of Internal Medicine of Hanwa-Senboku Hospital July, 1996 to June ,1997 | Patients ≥ 65 yo, current users of ACE-I only, or CCB only, and presented with pneumonia | Patients ≥ 65 yo current users of ACE-I only, or CCB only, without pneumonia | Ratio 1:4 age, sex | 275 | 81.1 (7.7) | Pneumonia | The hospital computerized pharmacy database | The CDC definition of nosocomial pneumonia and an abnormal chest radiograph was required | Age, Gender, COPD, Antacid |

Abbreviations: ACE-I, angiotensin-converting enzyme; ARB, angiotensin 2 receptor blocker; HTN, hypertension; DM, diabetes mellitus; CKD, chronic kidney disease; COPD, chronic obstructive pulmonary disease; CHF, congestive heart failure; SD,   standard deviation; NR, not reported; yo, year-old; ICD, International Classification of Diseases; ICPC, International Classification of Primary Care; CDC, Centers for Disease Control and Prevention; CAP, Community acquired pneumonia; ATC, Anatomical Therapeutic Chemical Classification; NIHSS, National Institutes of Health Stroke Scale

Note: We considered “de Groot 2014” as five different studies because it used five different datasets.

| **Supplementary Table S8: Risk of bias table for risk of pneumonia within RCTs** | | | | | | |
| --- | --- | --- | --- | --- | --- | --- |
| Study | D1 | D2 | D3 | D4 | D5 | Overall |
| Lee 2015 | Low | Some concerns | High | Low | Some concerns | High |
| Hou 2006 | Low | Low | Low | High | Some concerns | High |
| Ohkubo 2004 | Low | Low | Low | Low | Low | Low |
| Willenheimer 2002 | Some concerns | Low | Low | High | Some concerns | High |
| The GISEN Group 1997 | Low | Low | Low | High | Some concerns | High |
| Widimský 1995 | Some concerns | Low | Low | High | Some concerns | High |
| Køber 1995 | Low | Low | Low | High | Some concerns | High |
| Risk of bias domains: D1: Bias arising from the randomization process D2: Bias due to deviations from intended intervention D3: Bias due to missing outcome data D4: Bias in measurement of the outcome D5: Bias in selection of the reported result | | | | | | |

| **Supplementary Table S9: Risk of bias table for mortality within RCTs** | | | | | | |
| --- | --- | --- | --- | --- | --- | --- |
| Study | D1 | D2 | D3 | D4 | D5 | Overall |
| Lee 2015 | Low | Some concerns | High | Low | Some concerns | High |
| Hou 2006 | Low | Low | Low | Low | Low | Low |
| Ohkubo 2004 | Low | Low | Low | Low | Low | Low |
| Willenheimer 2002 | Some concerns | Low | Low | Low | Some concerns | Some concerns |
| The GISEN Group 1997 | Low | Low | Low | Low | Low | Low |
| Widimský 1995 | Some concerns | Low | Low | Low | Some concerns | Some concerns |
| Køber 1995 | Low | Low | Low | Lpw | Low | Low |
| Risk of bias domains: D1: Bias arising from the randomization process D2: Bias due to deviations from intended intervention D3: Bias due to missing outcome data D4: Bias in measurement of the outcome D5: Bias in selection of the reported result | | | | | | |

| **Supplementary Table S10: Risk of bias table for risk of withdrawal due to adverse effects within RCTs** | | | | | | |
| --- | --- | --- | --- | --- | --- | --- |
| Study | D1 | D2 | D3 | D4 | D5 | Overall |
| Lee 2015 | Low | Low | Some concerns | High | Some concerns | High |
| Hou 2006 | Low | Low | Low | High | Some concerns | High |
| Ohkubo 2004 | Low | Low | High | High | Some concerns | High |
| Willenheimer 2002 | Some concerns | Low | Low | High | Some concerns | High |
| The GISEN Group 1997 | Low | Low | Low | High | Some concerns | High |
| Køber 1995 | Low | Low | High | High | Some concerns | High |
| Risk of bias domains: D1: Bias arising from the randomization process D2: Bias due to deviations from intended intervention D3: Bias due to missing outcome data D4: Bias in measurement of the outcome D5: Bias in selection of the reported result | | | | | | |

| **Supplementary Table S11: Risk of bias table for swallowing function within RCTs** | | | | | | |
| --- | --- | --- | --- | --- | --- | --- |
| Study | D1 | D2 | D3 | D4 | D5 | Overall |
| Lee 2015 | Low | High | High | Low | Some concerns | High |
| Risk of bias domains: D1: Bias arising from the randomization process D2: Bias due to deviations from intended intervention D3: Bias due to missing outcome data D4: Bias in measurement of the outcome D5: Bias in selection of the reported result | | | | | | |

| **Supplementary Table S12: Newcastle–Ottawa Scale for risk of pneumonia within cohort studies** | | | | | | | | | | |
| --- | --- | --- | --- | --- | --- | --- | --- | --- | --- | --- |
| Study | Selection | | | | Comparability | | Outcome | | |  |
|  | Representativeness of the exposed cohort | Selection of non-exposed cohort | Ascertainment of exposure | Demonstration that outcome of interest was not present at start of study | Adjust for the most important risk factors | Adjust for other risk factors | Assessment of outcome | Follow-up length | Loss to follow-up rate | Total quality score |
| Davis 2020 | 0 | 1 | 1 | 1 | 0 | 0 | 1 | 1 | 1 | 6 |
| Kumazawa 2019 | 0 | 1 | 0 | 1 | 0 | 0 | 1 | 1 | 0 | 4 |
| Lai 2018 | 0 | 1 | 0 | 1 | 0 | 0 | 1 | 1 | 1 | 5 |
| Soto 2017-2 | 0 | 1 | 0 | 1 | 0 | 0 | 1 | 0 | 0 | 3 |
| Soto 2017-1 | 0 | 1 | 0 | 1 | 0 | 0 | 1 | 1 | 0 | 4 |
| Bang 2017 | 0 | 1 | 0 | 1 | 0 | 0 | 1 | 0 | 0 | 3 |
| Bang 2016 | 0 | 1 | 0 | 1 | 0 | 0 | 1 | 0 | 0 | 3 |
| Bang 2015 | 0 | 1 | 0 | 1 | 0 | 0 | 1 | 0 | 0 | 3 |
| Ishifuji 2017 | 0 | 1 | 0 | 1 | 0 | 0 | 1 | 1 | 0 | 4 |
| Chang 2015 | 1 | 1 | 0 | 1 | 0 | 0 | 1 | 0 | 1 | 5 |
| Wang 2015 | 0 | 1 | 0 | 1 | 0 | 0 | 1 | 1 | 1 | 5 |
| Shah 2014 | 1 | 1 | 0 | 1 | 1 | 0 | 1 | 1 | 1 | 7 |
| Alsumrain 2013 | 0 | 1 | 0 | 1 | 0 | 0 | 1 | 0 | 0 | 3 |
| Sato 2013 | 0 | 1 | 0 | 1 | 0 | 0 | 0 | 1 | 0 | 3 |
| Cuifang 2010 | 0 | 1 | 0 | 1 | 0 | 0 | 0 | 0 | 0 | 2 |
| Harada 2006 | 0 | 1 | 0 | 1 | 0 | 0 | 1 | 1 | 0 | 4 |
| Arai 2005 | 0 | 1 | 0 | 1 | 0 | 0 | 1 | 1 | 0 | 4 |
| Shibuya 2002 | 0 | 1 | 0 | 1 | 0 | 0 | 1 | 1 | 0 | 4 |
| Arai 2001 | 0 | 1 | 0 | 1 | 0 | 0 | 0 | 1 | 0 | 3 |
| Arai 2000 | 0 | 1 | 0 | 1 | 0 | 0 | 0 | 1 | 0 | 3 |
| Teramoto 1999 | 0 | 1 | 0 | 1 | 0 | 0 | 0 | 1 | 0 | 3 |
| Sekizawa 1998 | 0 | 1 | 0 | 1 | 0 | 0 | 1 | 1 | 0 | 4 |

| **Supplementary Table S13: Newcastle–Ottawa Scale for risk of pneumonia** **within case control studies** | | | | | | | | | | |
| --- | --- | --- | --- | --- | --- | --- | --- | --- | --- | --- |
| Study | Selection | | | | Comparability | | Exposure | | |  |
|  | Is the Case Definition Adequate? | Representativeness of the Cases | Selection of Controls | Definition of Controls | Adjust for the most important risk factors | Adjust for other risk factors | Ascertainment of Exposure | Same method of ascertainment for cases and controls | Non-Response Rate | Total quality score |
| Chhibber 2020 | 0 | 0 | 0 | 1 | 0 | 0 | 0 | 1 | 1 | 3 |
| Shah 2014-2 | 0 | 0 | 0 | 1 | 0 | 0 | 0 | 1 | 1 | 3 |
| de Groot 2014(LRGP) | 0 | 1 | 1 | 1 | 0 | 0 | 0 | 1 | 1 | 5 |
| de Groot 2014(AHC) | 0 | 1 | 1 | 1 | 0 | 0 | 0 | 1 | 1 | 5 |
| de Groot 2014(NPCD) | 0 | 1 | 1 | 1 | 0 | 0 | 0 | 1 | 1 | 5 |
| de Groot 2014(PH) | 0 | 1 | 1 | 1 | 0 | 0 | 0 | 1 | 1 | 5 |
| de Groot 2014(ANT) | 1 | 1 | 0 | 1 | 0 | 0 | 0 | 1 | 1 | 5 |
| Dublin, 2012 | 1 | 1 | 0 | 1 | 0 | 0 | 0 | 1 | 1 | 5 |
| Pope 2012 | 1 | 0 | 0 | 1 | 0 | 0 | 0 | 0 | 0 | 2 |
| Vilanova 2012 | 0 | 1 | 1 | 0 | 0 | 0 | 0 | 0 | 0 | 2 |
| Mukamal 2010 | 0 | 0 | 1 | 1 | 0 | 0 | 0 | 1 | 1 | 4 |
| Myles 2009 | 0 | 1 | 1 | 1 | 1 | 0 | 0 | 1 | 1 | 6 |
| Marciniak 2009 | 1 | 0 | 0 | 1 | 0 | 0 | 0 | 1 | 1 | 4 |
| van de Garde 2007 | 0 | 0 | 1 | 1 | 0 | 0 | 0 | 1 | 1 | 4 |
| van de Garde 2006 | 0 | 1 | 1 | 1 | 0 | 0 | 0 | 1 | 1 | 5 |
| Etminan 2006 | 0 | 0 | 1 | 1 | 1 | 0 | 0 | 1 | 1 | 5 |
| Takahashi 2005 | 1 | 1 | 0 | 1 | 0 | 0 | 0 | 1 | 1 | 5 |
| Ohse 2004 | 0 | 0 | 0 | 1 | 0 | 0 | 0 | 1 | 1 | 3 |
| El Solh 2004 | 1 | 0 | 0 | 1 | 0 | 0 | 0 | 1 | 1 | 4 |
| Okaishi 1999 | 1 | 0 | 0 | 1 | 0 | 0 | 0 | 1 | 1 | 4 |

| **Supplementary Table S14: Newcastle–Ottawa Scale for mortality within cohort studies** | | | | | | | | | | |
| --- | --- | --- | --- | --- | --- | --- | --- | --- | --- | --- |
| Study | Selection | | | | Comparability | | Outcome | | |  |
|  | Representativeness of the exposed cohort | Selection of non-exposed cohort | Ascertainment of exposure | Demonstration that outcome of interest was not present at start of study | Adjust for the most important risk factors | Adjust for other risk factors | Assessment of outcome | Follow-up length | Loss to follow-up rate | Total quality score |
| Davis 2020 | 0 | 1 | 1 | 1 | 0 | 0 | 1 | 1 | 1 | 6 |
| Lai 2018 | 0 | 1 | 0 | 1 | 0 | 0 | 1 | 1 | 1 | 5 |
| Chang 2015 | 1 | 1 | 0 | 1 | 0 | 0 | 1 | 0 | 1 | 5 |
| Cuifang 2010 | 0 | 1 | 0 | 1 | 0 | 0 | 0 | 0 | 0 | 2 |

| **Supplementary Table S15: The result of subgroup analysis and sensitivity analysis of the risk of pneumonia with use of angiotensin-converting enzyme inhibitors (ACE-I) compared with control treatment among RCTs, cohort, and case-control studies** | | | | | | | |  |
| --- | --- | --- | --- | --- | --- | --- | --- | --- |
|  |  |  |  | Number of Study | Pooled odds Ratio^*^ | 95% CI | P for interaction |  |
| RCT | | | |  |  |  |  |  |
|  | Subgroup analysis | | |  |  |  |  |  |
|  |  | Ethnicity | |  |  |  | 0.90 |  |
|  |  |  | Asian | 3 | 0.83 | 0.30–2.27 |  |  |
|  |  |  | Non Asian | 5 | 0.77 | 0.59–1.01 |  |  |
|  |  | Age | |  |  |  | 0.21 |  |
|  |  |  | ≥ 65 y | 1 | 1.51 | 0.59–3.86 |  |  |
|  |  |  | < 65 y | 4 | 0.81 | 0.63–1.03 |  |  |
|  |  | Observation period | |  |  |  | 0.33 |  |
|  |  |  | < 3months | 1 | 0.73 | 0.03–18.18 |  |  |
|  |  |  | ≥ 3months and < 24 months | 3 | 1.43 | 0.60–3.41 |  |  |
|  |  |  | ≥ 24 months | 3 | 0.72 | 0.58–0.91 |  |  |
|  | Sensitivity Analysis | | |  |  |  |  |  |
|  |  | Exclusion of imputed studies | | 4 | 0.82 | 0.47–1.44 |  |  |
|  |  | Exclusion of inappropriate definition of pneumonia | | 1 | 1.51 | 0.59–3.86 |  |  |
| Cohort | | | |  |  |  |  |  |
|  | Subgroup analysis | | |  |  |  |  |  |
|  |  | Ethnicity | |  |  |  | 0.02 |  |
|  |  |  | Asian | 17 | 0.68 | 0.56–0.83 |  |  |
|  |  |  | Non Asian | 20 | 0.89 | 0.80–0.99 |  |  |
|  |  | Age | |  |  |  | <0.001 |  |
|  |  |  | ≥ 65 y | 9 | 0.65 | 0.52–0.81 |  |  |
|  |  |  | < 65 y | 18 | 1.03 | 0.94–1.12 |  |  |
|  |  | Observation period | |  |  |  | <0.001 |  |
|  |  |  | < 3months | 1 | 1.11 | 1.05–1.18 |  |  |
|  |  |  | ≥ 3months and < 24 months | 4 | 0.66 | 0.38–1.15 |  |  |
|  |  |  | ≥ 24 months | 11 | 0.71 | 0.57–0.88 |  |  |
|  |  | History of stroke | |  |  |  | 0.05 |  |
|  |  |  | History of stroke + | 10 | 0.52 | 0.30–0.93 |  |  |
|  |  |  | History of stroke - | 6 | 0.94 | 0.81–1.10 |  |  |
|  |  | History of neurodegenerative disease | |  |  |  | 0.05 |  |
|  |  |  | History of neurodegenerative disease + | 1 | 0.78 | 0.63–0.95 |  |  |
|  |  |  | History of neurodegenerative disease - | 1 | 1.06 | 0.84–1.34 |  |  |
|  | Sensitivity analysis | | |  |  |  |  |  |
|  |  | Exclusion of univariate studies | | 22 | 0.92 | 0.84–1.01 |  |  |
|  |  | Exclusion of imputed studies | | 21 | 0.86 | 0.77–0.95 |  |  |
|  |  | Exclusion of inappropriate definition of pneumonia | | 10 | 0.51 | 0.29–0.90 |  |  |
|  |  | Exclusion of studies which examined for pneumonia only in inpatient or outpatient setting | | 13 | 0.85 | 0.73–0.99 |  |  |
| *Pooled by the DerSimonian-Laird random-effects model.  Abbreviation: CI, confidence interval; RCT, randomized control study. | | | | | | | |  |
|  |  |  |  |  |  |  |  |  |

**Supplementary Figure S1: PRISMA Flow Diagram**

Studies excluded, with reason(n=1)

Protocols without results (n=1)

Studies excluded, with reasons (n=7)

Abstracts of other included studies (n=5)

Same studies of other included studies (n=2)

Full-text articles assessed for eligibility
(n = 54)

Full-text articles excluded, with reasons
(n =44)

Wrong intervention or comparison (n=7)

Wrong population (n=3)

Wrong outcome(n=16)

Wrong study design (n=18)

Records excluded
(n =1877)

Studies included in qualitative synthesis
(n = 46)

(47 Records)

**Included**

Studies included in quantitative synthesis (meta-analysis)
(n = 45)

(46 Records)

Records identified through database searching
total (n =1452)

MEDLINE via PubMed (n=271)

EMBASE (n=1150)

CENTRAL (n=31)

Additional records identified through other sources

total (n=2172)

Clinical Trials(n=8)

ICTRP (n=2164)

Guidelines (n =1)

Records after duplicates removed
(n =1962)

**Identification**

**Eligibility**

Additional records identified through citation search (n=13)

Records screened
(n =98)

**Screening**

**Supplementary Figure S2: Mortality with use of angiotensin converting enzyme inhibitors (ACE-I) compared with control treatment among RCTs**


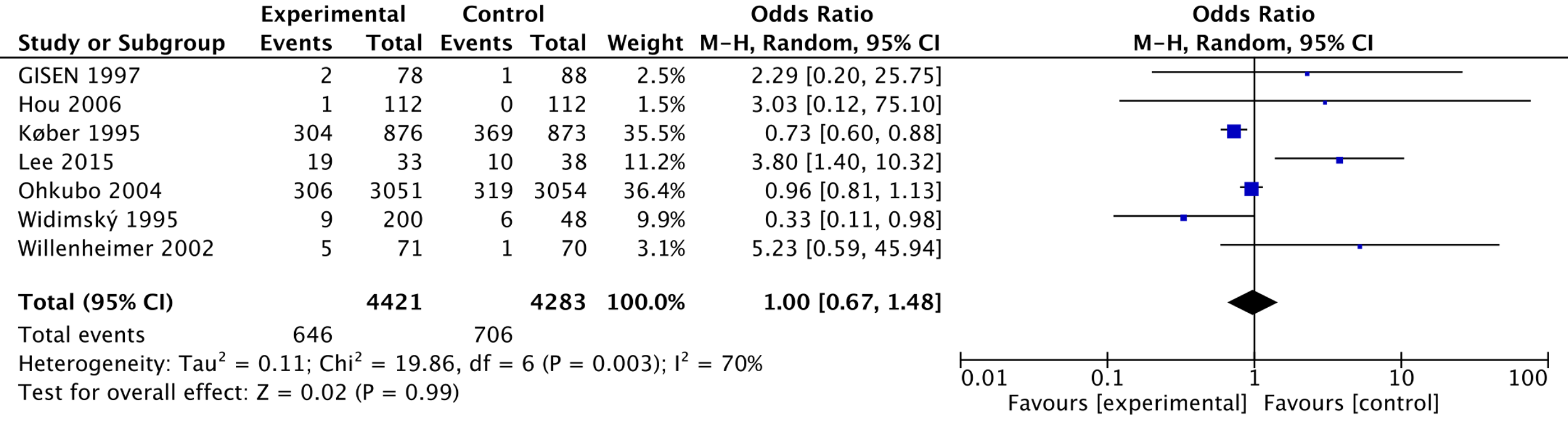


**Supplementary Figure S3: Mortality with use of angiotensin converting enzyme inhibitors (ACE-I) compared with control treatment among cohort and case control studies**


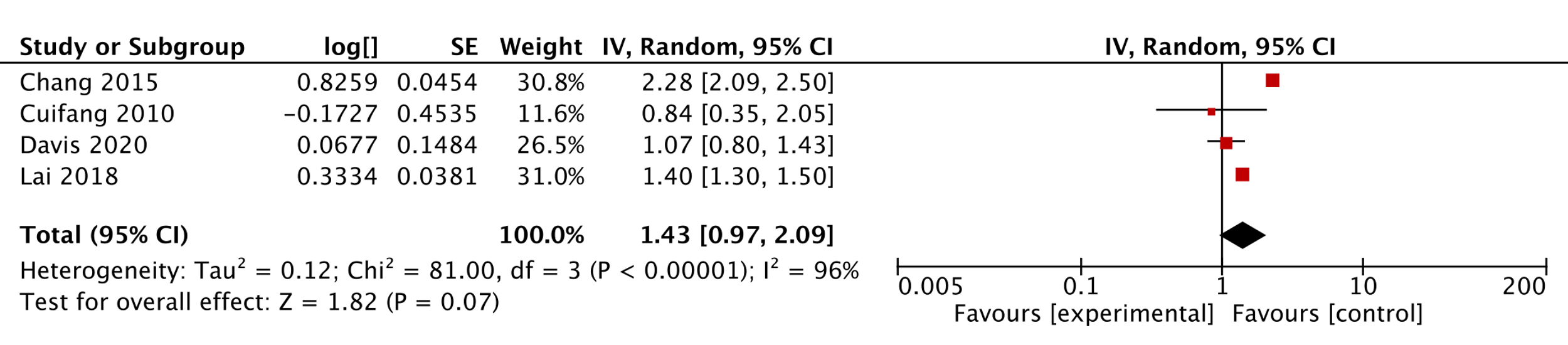


We used adjusted OR if it was available and used an unadjusted OR if it was not.

**Supplementary Figure S4: Withdrawal due to adverse effects with use of angiotensin converting enzyme inhibitors (ACE-I) compared with control treatment among RCTs**


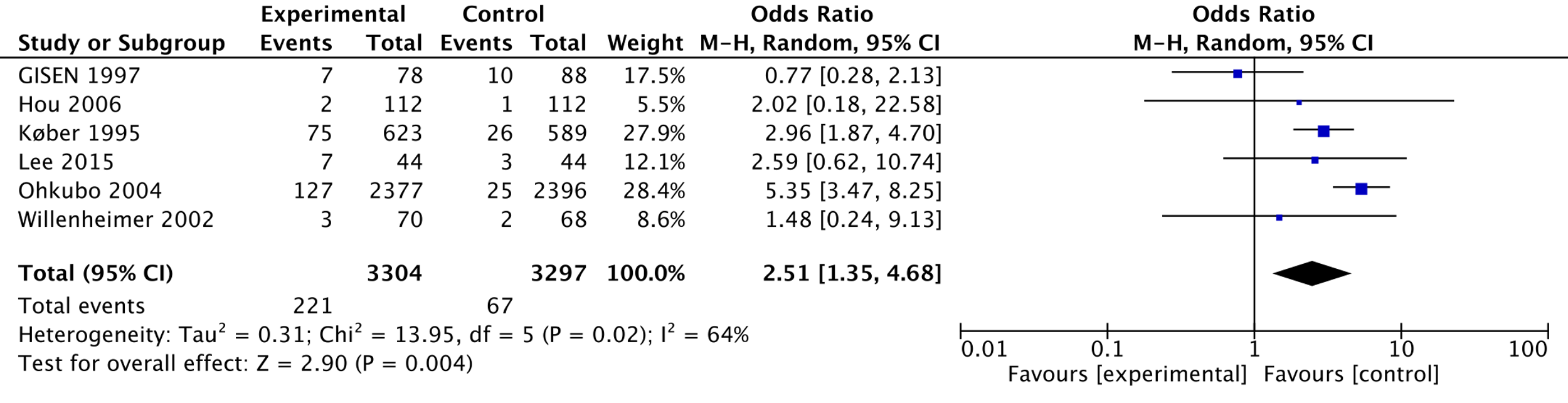

Supplement: Supplementary file 1 — App S1 [file JGF2-23-217-s001.docx]
